# Assessment of land subsidence and future flood hazards for coastal cities of the Gulf of Mexico using time series InSAR

**DOI:** 10.1007/s11069-026-08203-9

**Published:** 2026-05-14

**Authors:** Yuxin Yuan, Lei Wang

**Affiliations:** Department of Civil and Architectural Engineering and Construction Management, University of Cincinnati, Cincinnati, OH 45221 USA

**Keywords:** Land subsidence, SBAS-InSAR, Flood, Gulf of Mexico

## Abstract

Sea level rise poses a significant natural hazard to coastal cities, with profound socio-economic and environmental impacts. This study analyzes new measurements of ongoing subsidence in Houston, New Orleans, and Tampa using Sentinel-1A SAR data (2019–2024) and evaluates its impact on future flooding risks. While the overall surface in these cities remains relatively stable, this study has identified multiple previously unrecognized land deformation zones. InSAR time-series deformation rates in the line-of-sight (LOS) direction range from − 42 to 10 mm/year in Houston, − 48 to 19 mm/year in New Orleans, and − 144 to 24 mm/year in Tampa. InSAR-derived velocities are correlated with land cover, with higher subsidence rates observed in vegetated areas compared to developed regions. Geographically weighted regression (GWR) is applied to quantify the spatially varying relationships between subsidence and potential driving factors. The results indicate that both groundwater withdrawal and hydrocarbon-related activities contribute to the observed deformation patterns, although their influence varies across locations. To assess future flood risk, InSAR-derived deformation data are integrated with the SSP 5–8.5 sea level rise scenario. The results show that subsidence increases the inundation area over sea level rise alone by up to 86% in Houston, 5% in New Orleans, and 50% in Tampa. These findings provide valuable insights for disaster risk management and urban planning in coastal regions.

## Introduction

As global climate change accelerates, the rate of sea level rise is increasing annually, with projections indicating a substantial escalation in the coming decades (Catalao et al. 2020). According to the Intergovernmental Panel on Climate Change (IPCC) report, even under the lowest emission scenario (SSP1-1.9), global sea levels are expected to rise by 0.28–0.55 m by 2100 relative to 1995–2014 (IPCC 2023). The rising sea level, combined with climate factors such as intensified storms and wave action, poses severe threats to coastal infrastructure (Befus et al. 2020; Buzzanga et al. 2023), coastal erosion (Vousdoukas et al. 2022; Weisse et al. 2021), freshwater salinization (Jeppesen et al. 2020; Paldor and Michael 2021), and loss of terrestrial habitat (McMichael et al. 2020). Additionally, low-lying coastal areas, such as the Gulf of Mexico, are susceptible to accelerating rates of sea level rise, especially in areas experiencing high rates of land subsidence (Bhatia et al. 2019; Karegar et al. 2020; Shirzaei et al. 2021), profoundly exacerbating flooding hazards and impacting local communities and economic activities (Miller and Shirzaei 2021).

Previous studies on coastal flood risk have utilized Global Positioning System (GPS) surface deformation data to evaluate land subsidence rates (Irawan et al. 2021; Karegar et al. 2020; Yuwono et al. 2024). However, the spatial coverage of GPS permanent stations is limited (Ansari et al. 2018), and their establishment and maintenance are labor-intensive, costly, and time-consuming (Li et al. 2022; van Natijne et al. 2022). Interferometric Synthetic Aperture Radar (InSAR) is widely used in landslides (Bekaert et al. 2020; Zhou et al. 2022; Zhang et al. 2022), earthquakes (Li et al. 2021; Liu et al. 2021), and land subsidence (Cigna and Tapete 2021; Xiao et al. 2022) due to high accuracy, high resolution, and all-weather advantages, providing significant technical support for surface deformation monitoring. By processing SAR images, it is possible to quickly obtain deformation information over a large area, especially for regional surface deformation monitoring. SBAS-InSAR utilizes multiple interferometric pairs with small temporal and spatial baselines to reduce decorrelation and atmospheric noise, enabling reliable retrieval of slow deformation signals over large areas. Compared to traditional InSAR approaches, SBAS-InSAR provides higher spatial coverage and more stable time-series deformation estimates, making it especially suitable for regional-scale subsidence monitoring (Li et al. 2022). Given the spatial extent of the Gulf Coast cities and the need for consistent multi-year deformation analysis, SBAS-InSAR is well-suited for this study.

Considerable work has been done to quantify land subsidence in the Gulf of Mexico, particularly in major coastal cities such as Houston and New Orleans, where subsidence has been extensively documented using geodetic and InSAR-based approaches. Previous studies have revealed pronounced spatial variability in subsidence patterns and identified key driving mechanisms, including groundwater extraction, hydrocarbon production, and sediment compaction (Jones et al. 2016; Karegar et al. 2020; Shirzaei et al. 2021). In deltaic environments such as the Mississippi River Delta, subsidence is strongly controlled by the compaction of Holocene sediments, with rates increasing toward areas with thicker sediment deposits (Deng 2025).

Recent advances in satellite-based InSAR have significantly improved the spatial resolution and temporal continuity of subsidence monitoring. Qu et al. (2023) used data from the Advanced Land Observation Satellite (ALOS) between 2007 and 2011 to monitor subsidence along the Gulf Coast. Their findings reveal that subsidence is primarily driven by petroleum reservoir depressurization and aquifer compaction due to groundwater extraction. A study undertaken by Wang et al. (2024) mapped the average land subsidence rate (2017–2020) from Sentinel-1 data, finding that even slight subsidence can significantly enhance hurricane-induced flooding and impact passive flood mapping. Furthermore, Nur et al. (2024) analyzed data obtained from the Sentinel-1 to quantitatively assess the extent and rate of land subsidence in southeast Texas. In particular, recent InSAR-based investigations have revealed strong spatial heterogeneity in subsidence within urban coastal environments and highlighted its implications for flood risk. For example, localized subsidence exceeding 20 mm/year has been identified in parts of the Greater New Orleans area, with potential consequences for the long-term performance of flood protection infrastructure in low-lying urban and wetland settings (Fiaschi et al. 2025). Similarly, studies in the Houston–Galveston region have shown that spatially variable vertical land motion, when combined with sea-level rise, can substantially amplify flood exposure and increase infrastructure vulnerability (Buzzanga et al. 2025). Beyond regional studies, large-scale assessments have further emphasized the widespread nature of land subsidence and its societal impacts. Nationwide InSAR-based mapping across the United States has revealed that subsidence affects extensive areas and poses risks to millions of people and critical infrastructure (Xiong et al. 2026). In addition, recent studies have begun to integrate InSAR-derived subsidence with predictive modeling frameworks to assess the coupled effects of subsidence and sea-level rise on future inundation in deltaic environments (Mondal et al. 2026).

The cities along the Gulf of Mexico are particularly vulnerable to flooding risks due to rising sea levels, storm surges, and subsidence. In particular, Houston is at significant risk of flooding exacerbated by subsidence, while New Orleans’ low-lying areas, protected by levees and pumps, remain prone to inundation. Similarly, Tampa experienced severe flooding in 2024 as Hurricane Milton brought intense rainfall and widespread damage.

This study aims to assess future flood risks in these regions by analyzing the combined effects of land subsidence and sea level rise. Using InSAR technology, we examine land subsidence from January 2019 to January 2024, providing high-resolution data on surface displacement. By integrating these data with sea level rise projections and Digital Elevation Models (DEMs), we predict areas at risk of inundation by 2100. This research will offer valuable insights into the spatial extent of flooding hazards and inform future resilience planning, flood management, and mitigation strategies in these vulnerable coastal cities.

## Methods and materials

### Geological and hydrogeological setting

**Fig. 1:**
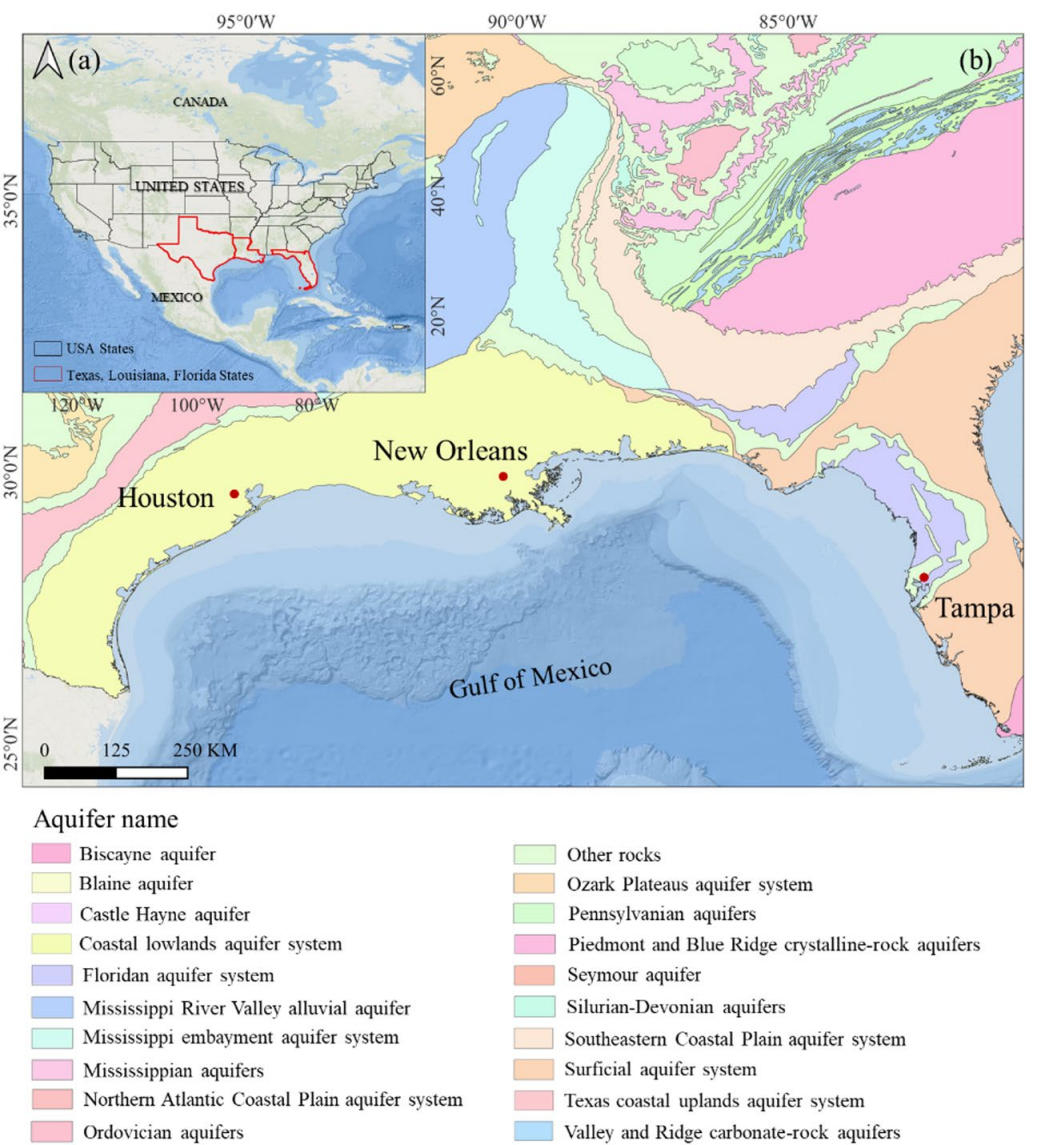
**a** Location of study area **b** Main aquifers in the coastal cities of the Gulf of Mexico

The study area encompasses three major coastal U.S. cities along the Gulf of Mexico, including Houston, New Orleans, and Tampa, which are situated above the Coastal Lowlands Aquifer System and the Floridan Aquifer System (Fig. 1).

Houston is underlain by the Gulf Coast Aquifer System, along the Gulf of Mexico from Louisiana to Mexico, including Chicot, Evangeline, and Jasper aquifers, as shown in Table 1. The Chicot Aquifer is the shallowest and consists of Holocene and Pleistocene-age sediments. The underlying Evangeline aquifer consists of Pliocene and Miocene-age sediments, with no confining unit between the two aquifers. The Chicot Aquifer outcrops inland and extends southeastward, transitioning into the deeper Evangeline and Jasper aquifers. The Jasper Aquifer is formed from Miocene-age sediments (Baker 1979). These sediments were deposited from the Miocene to the Holocene and thicken toward the Gulf of Mexico. In Houston, thick sequences of undercompacted coastal and deltaic sediments are associated with the Gulf Coastal Plain, where subsidence is influenced by both sediment compaction and anthropogenic activities such as groundwater withdrawal and hydrocarbon extraction (Haley et al. 2021). These sediments are typically composed of interbedded sand, silt, and clay layers, with clay-rich units being particularly susceptible to compaction due to their high compressibility. In addition, changes in pore pressure associated with fluid withdrawal can accelerate compaction processes, further contributing to observed ground deformation.

**Table 1:** Aquifers in the study area

Houston aquifer system	New Orleans aquifer system	Tampa aquifer system
Evangeline aquifer system	Gramercy aquifer	Surficial aquifer system
Catahoula confining system	Norco aquifer	Upper confining unit
Jasper aquifer	Gonzales-New Orleans aquifer	
Burkville confining system	“1,200-foot” sand (aquifer)	Floridan aquifer system
Chicot aquifer		

New Orleans, extending from St. Charles Parish to Orleans Parish, encompasses the Gramercy, Norco, Gonzales-New Orleans, and 1200 Foot Sand aquifer (Dial and Tomaszewski 1988). Under it is the Norco Aquifer, most of which is in Jefferson Parish but becomes thin or absent in western Orleans Parish north of the Mississippi River. It consists primarily of fine to coarse sand with occasional fine gravel. The Gonzales-New Orleans Aquifer is the most laterally extensive unit in the region, underlying both Jefferson and Orleans parishes. Composed mainly of fine to medium sand with a relatively uniform texture, it dips southward, with its upper boundary ranging from approximately 400 feet below NGVD 29 in the northeast to 700 feet in the south. Its thickness varies from 150 to 250 feet north of the Mississippi River and can reach up to 300 feet in the southern region (Rollo 1966; Tomaszewski 2003). In New Orleans, the subsurface is dominated by young, fine-grained deltaic deposits associated with the Mississippi River Delta, including silts, clays, and organic-rich materials such as peat (Törnqvist et al. 2008). These materials are highly compressible and prone to both mechanical compaction and biochemical degradation. In particular, peat-rich layers can undergo volume reduction through compaction, oxidation, and irreversible shrinkage, all of which contribute to land subsidence. As a result, subsidence in this region is largely controlled by widespread consolidation of these soft sediments, leading to relatively uniform deformation across the study area.

In Tampa, there are three primary aquifer units: the Surficial aquifer system (SAS), upper confining unit (UCU), and Floridian aquifer system (FAS) (Miller 1986; Williams and Kuniansky 2015a, b). The Surficial Aquifer (SA) is a shallow, unconfined system composed mainly of sand, sandy clay, and marl. Its thickness varies across the region, ranging from approximately 25 ft in the northern Tampa Bay to 250 ft in the southern bay (Hutchinson 1992). The UCU underlies the SAS and consists of lower-permeability sediments, restricting water exchange between the SAS and the deeper FAS (Williams and Kuniansky 2015a, b). It contains multiple water-bearing zones separated by confining layers. Its thickness increases from 25 ft in Old Tampa Bay to approximately 250 ft in the southern bay, but in certain locations, erosion or anthropogenic activities have breached the UCU, directly exposing the FAS to surface influences (Hutchinson 1992). UCU is present throughout much of Florida, but is not present everywhere in the study area. The FAS, a regional carbonate aquifer, is the most significant groundwater source in the area, primarily composed of Miocene to Oligocene limestone formations (Tampa Member, Suwannee Limestone, Ocala Limestone, and Avon Park Formation), with an average thickness of 1100 ft (Hutchinson 1992; Williams and Kuniansky 2015a, b). Compared to LFA, the UFA is more productive and becomes a major source of drinking water in the region, primarily made up of permeable limestone and dolostone (Ferguson and Hampton 2021). In Tampa, the subsurface is primarily underlain by relatively stable carbonate formations associated with the Florida Platform, which consists mainly of limestone and other marine sedimentary rocks. Compared to the unconsolidated sediments of the Gulf Coast and Mississippi Delta, these carbonate units generally exhibit lower compressibility, which limits large-scale subsidence driven by sediment compaction. Subsidence in this area is generally more localized and is often associated with groundwater extraction from shallow aquifer systems. Previous studies have reported subsidence on the order of a few millimeters per year in areas underlain by Pleistocene and Holocene sediments, where groundwater withdrawal can induce compaction of unconsolidated layers (Hutchinson 1992).

### Dataset

#### SAR data

Sentinel-1 SAR data from 2019 to 2024 were used for time-series deformation analysis. Interferometric pairs were generated using the HyP3 platform provided by the Alaska Satellite Facility (ASF). HyP3 performs interferometric processing on Sentinel-1 single-look complex (SLC) data, including co-registration of SAR images, interferogram formation, removal of the topographic phase using an external DEM, adaptive filtering, and phase unwrapping using a minimum cost flow (MCF) algorithm. The resulting products include wrapped and unwrapped interferograms, coherence maps, amplitude images, and look vector information, which were used as inputs for subsequent time-series analysis. A total of 666 interferometric pairs for Houston, 497 for New Orleans, and 697 for Tampa, spanning from January 2019 to January 2024, were utilized to derive land deformation. The multilook factor was set to 10:2, the temporal baseline was 60 days, and the spatial baseline was 200 m.

#### Digital elevation model

In this study, we utilized a digital elevation model (DEM) with a resolution of 30 m per pixel, obtained from the Shuttle Radar Topography Mission (SRTM) data.

#### Validation based on GPS data

The GPS data for 2019–2024 were provided by the National Geodetic Survey (NGS) and processed by the Nevada Geodetic Laboratory with respect to the IGS14 reference frame.

#### National land cover data

The 2023 National Land Cover (NLC) Database, managed by USGS, provided the land cover data with 30-m resolution, including 15 categories such as water, developed areas, forests, grasslands, and wetlands. To integrate deformation velocity with the NLC data, we resampled the velocity data to match land cover types and simplified the classification into 8 categories: developed, barren land, forest, shrub/scrub, grassland, pasture/hay, cultivated crops, and wetlands.

#### Sea level rise data

Sea-level rise (SLR) projections are derived from the Shared Socioeconomic Pathways (SSPs) developed by IPCC. SSPs represent different socioeconomic trajectories, including population growth, economic development, and technological progress, influencing greenhouse gas emissions and climate change mitigation efforts. In this study, we adopt projections based on the SSP5-8.5 scenario for the year 2100.

### SBAS-InSAR processing

SBAS-InSAR is a time series InSAR analysis method proposed by Bernardino (Berardino et al. 2002). The method generates interferometric image pairs based on a shorter spatio-temporal baseline criterion and utilizes Singular Value Decomposition (SVD) to solve the surface deformation time series of a certain time period in the study area.

In the time period $\left( {t_{0} ,t_{1} , \ldots ,t_{n} } \right)$, there are a total of $N + 1$ SAR images, and one of them is selected as the super-master image to generate $M$ differential interferograms by setting the proper temporal and spatial baseline thresholds, and $M$ is satisfied:

1 $$ \frac{N + 1}{2} \le M \le \frac{N(N + 1)}{2} $$

The interferometric phase of a certain image element $\left( {x,r} \right)$ on the ith differential interferogram generated at the moment of $t_{A}$ and $t_{B}$ can be expressed as follows if the residual terrain phase, atmospheric phase, and noise phase are not taken into account:

2 $$ \delta_{\varphi i} \left( {x,r} \right) = \varphi_{B} \left( {x,r} \right) - \varphi_{A} \left( {x,r} \right) \approx \frac{4\pi }{\lambda }\left[ {d\left( {t_{B} ,x,r} \right) - d\left( {t_{A} ,x,r} \right)} \right] $$

where $\lambda$ is the wavelength, $d\left( {t_{B} ,x,r} \right)$ and $d\left( {t_{A} ,x,r} \right)$ are the radar line-of-sight direction cumulative deformations at the moments $t_{A}$ and $t_{B}$, respectively, with respect to the initial moment.

For $M$ interferograms, $M$ equations can be obtained according to Eq. (2), and this system of equations can be expressed in terms of a matrix as:

3 $$ \delta_{\varphi } \left( {x,r} \right) = A\varphi \left( {x,r} \right) $$

where $A$ is a matrix of coefficients $M \times N$. $A$ is solved by least squares when the rank of $A$ is $N$:

4 $$ \Phi = (A^{T} A)^{ - 1} A^{T} \delta_{\varphi } $$

When the rank of $A$ is less than $N$, the phase value is replaced by the average rate of phase change between the two view images, and Eq. (4) can be rewritten as:

5 $$ Bv = \delta \varphi $$

where $B$ is the $M \times N$ matrix of coefficients and $v^{T}$ can be expressed as:

6 $$ v^{T} = \left[ {v_{1} = \frac{{\varphi_{1} }}{{t_{1} - t_{0} }}, \ldots ,v_{N} = \frac{{\varphi_{N} - \varphi_{N - 1} }}{{t_{N} - t_{N - 1} }}} \right] $$

The deformation rate of the study area is obtained by using the singular value decomposition method to calculate Eq. (6), and finally, by integrating the rate according to the period between the SAR images, the cumulative deformation in the corresponding period can be obtained. The generated interferograms were subsequently analyzed using the MintPy time-series InSAR framework. It was used to construct the deformation time series through a sequence of post-processing steps, including reference point selection, phase closure evaluation, correction of residual DEM-related phase components, and atmospheric delay mitigation (Yunjun et al. 2019).

## Results and analyses

### InSAR-derived deformation velocity

#### Spatiotemporal pattern of land subsidence

**Fig. 2:**
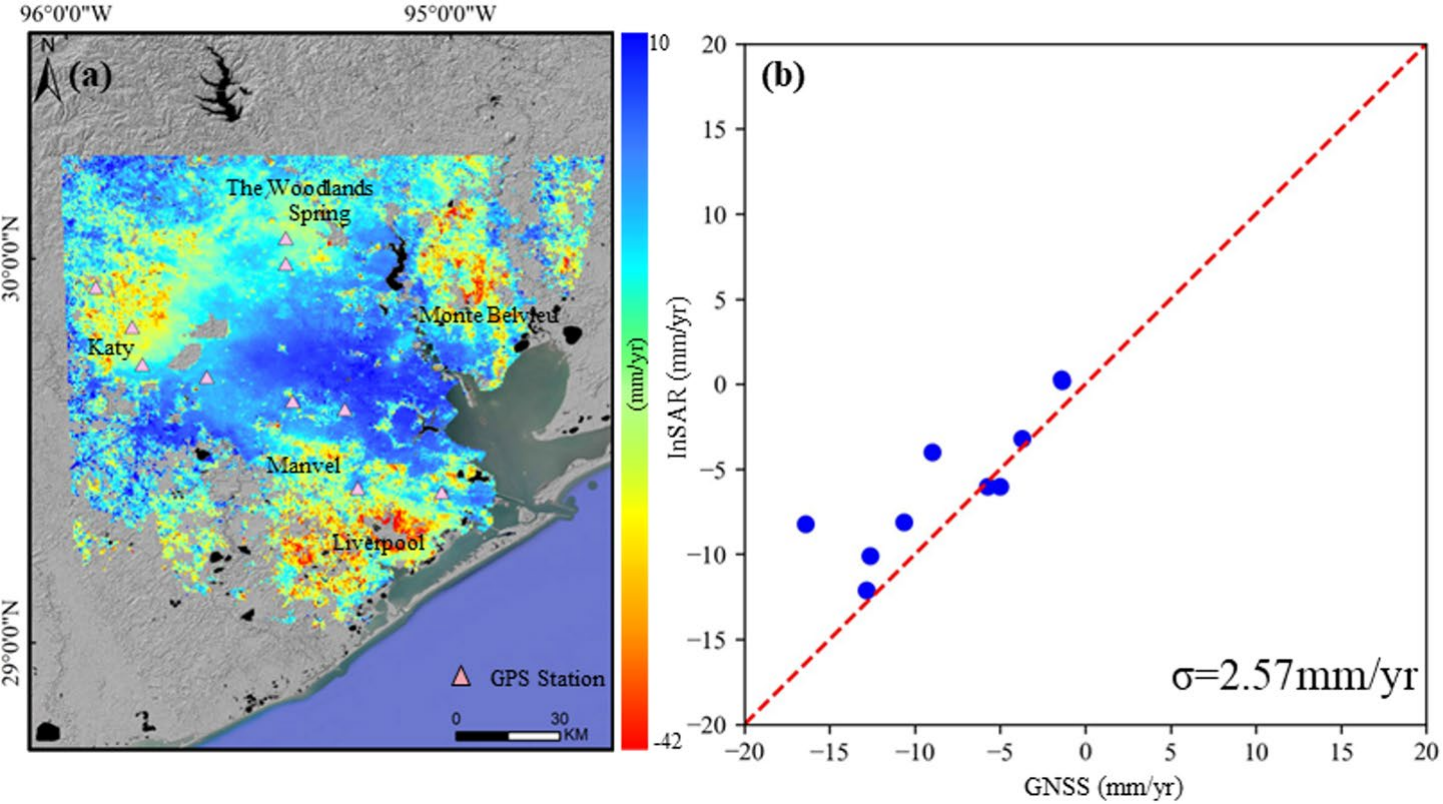
**a** A map of average land surface deformation derived from Sentinel 1A images (2019–2024) in Houston; **b** Comparison of InSAR-derived vertical subsidence velocity to GPS measurements

**Fig. 3:**
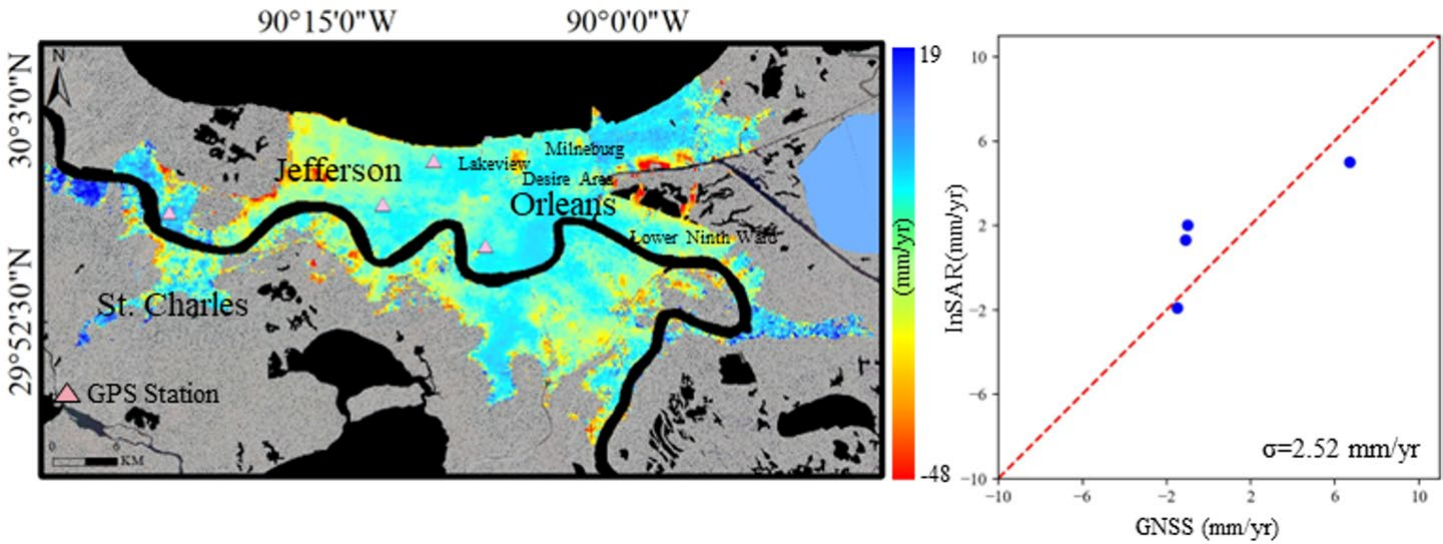
**a** A map of average land surface deformation derived from Sentinel 1A images (2019–2024) in New Orleans; **b** Comparison of InSAR-derived vertical subsidence velocity to GPS measurements

**Fig. 4:**
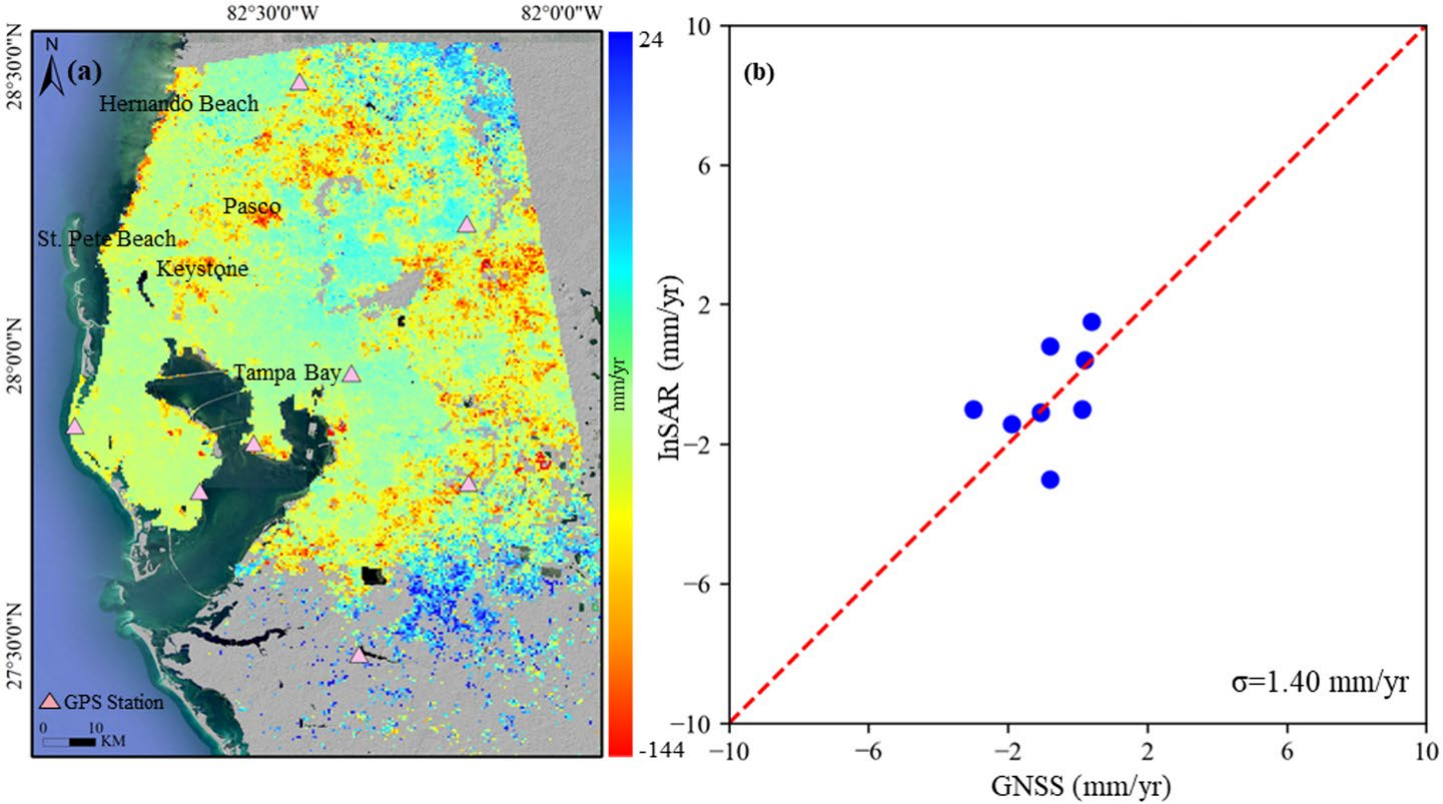
**a** A map of average land surface deformation derived from Sentinel 1A images (2019–2024) in Tampa; **b** Comparison of InSAR-derived vertical subsidence velocity to GPS measurements

The InSAR-derived deformation rates extracted from Sentinel-1 data during the period from January 2019 to January 2024 in Houston, New Orleans, and Tampa are shown in Figs. 2, 3 and 4.

Between 2019 and 2024, InSAR data captured a varied subsidence across Houston, with LOS velocities ranging from − 42 to 10 mm/year. While most of the city remains stable, four distinct subsidence areas have been identified. The most severe subsidence is found in the southern region, particularly around Liverpool, where the subsidence rate reaches as high as 20–36 mm/year. The next highest subsidence occurs in Katy, with land subsidence velocities ranging from 10 to 26 mm/year. Subsidence in the eastern zone, particularly in Monte Belvieu, is more localized, with rates reaching − 20 mm/year. Lower levels of subsidence are observed in the northern areas, such as Spring and The Woodlands, as well as in scattered spots in the south, like Manvel. Here, the deformation is less intense, typically within the range of − 5 to − 15 mm/year, and appears as smaller, fragmented subsidence bowls.

The map of land deformation in Fig. 3 reveals the subsidence pattern in New Orleans, with notable areas of significant subsidence. In particular, several parishes, such as St. Charles, Jefferson, and Orleans, demonstrate clear ground movement. In Orleans, especially in the Lower Ninth Ward, subsidence is particularly severe, reaching rates of about 40 mm/year. The northern part of Jefferson shows widespread but lower subsidence, with an average rate of 2 mm/year. However, in the western part of Jefferson, Louis Armstrong New Orleans International Airport is significantly affected, with subsidence rates up to 22 mm/year. Areas along riverbanks and wetlands show moderate and low subsidence, typically between 5 and 15 mm/year. In the southern parts of the city, subsidence is also present, though at lower rates (5–15 mm/year). These areas feature fragmented subsidence, with numerous subsidence bowls dispersed across various locations. Additionally, some northern coastal areas experience mild, widespread subsidence, which, combined with rising sea levels, could present long-term flood management and infrastructure challenges.

Figure 4 is the LOS rate map of Tampa, indicating that there are multiple subsidence areas. The eastern and southern regions experience the most severe subsidence, with rates between − 10 and − 30 mm/year. A large area in the northwest Tampa Bay region, particularly in Keystone and Pasco, includes a 30-km-long coastal section from St. Pete Beach to Hernando Beach subsiding at rates exceeding 5 mm/year in the LOS direction. Coastal regions also show notable subsidence, with most areas experiencing rates between − 5 and − 10 mm/year, and localized hotspots exhibiting even higher rates. In contrast, the central urban and western areas show lower subsidence rates, with some locations experiencing slight uplift, ranging from − 2 to 2 mm/year.

#### Validation

The vertical results transformed from LOS results using the local incidence angle were used for validation, assuming that the vertical component was approximated assuming that vertical motion dominates in subsidence-prone regions, where horizontal deformation is generally small (Deng 2025; Je Kim et al. 2026; Modeste et al. 2021; Zhong et al. 2022):

7 $$ V_{v} = \frac{{V_{LOS} }}{\cos (i)} $$

where $i$ is the angle of incidence at the considered pixels.

Figures 2, 3 and 4b shows the validation of InSAR-derived subsidence against the GPS measurements. The standard deviation of the difference between Sentinel-1A and GPS is 2.57 mm/year, 2.25 mm/year, and 1.40 mm/year in Houston, New Orleans, and Tampa, respectively, which constitutes good agreement.

### Subsidence exposure summarized by land-cover class

**Fig. 5:**
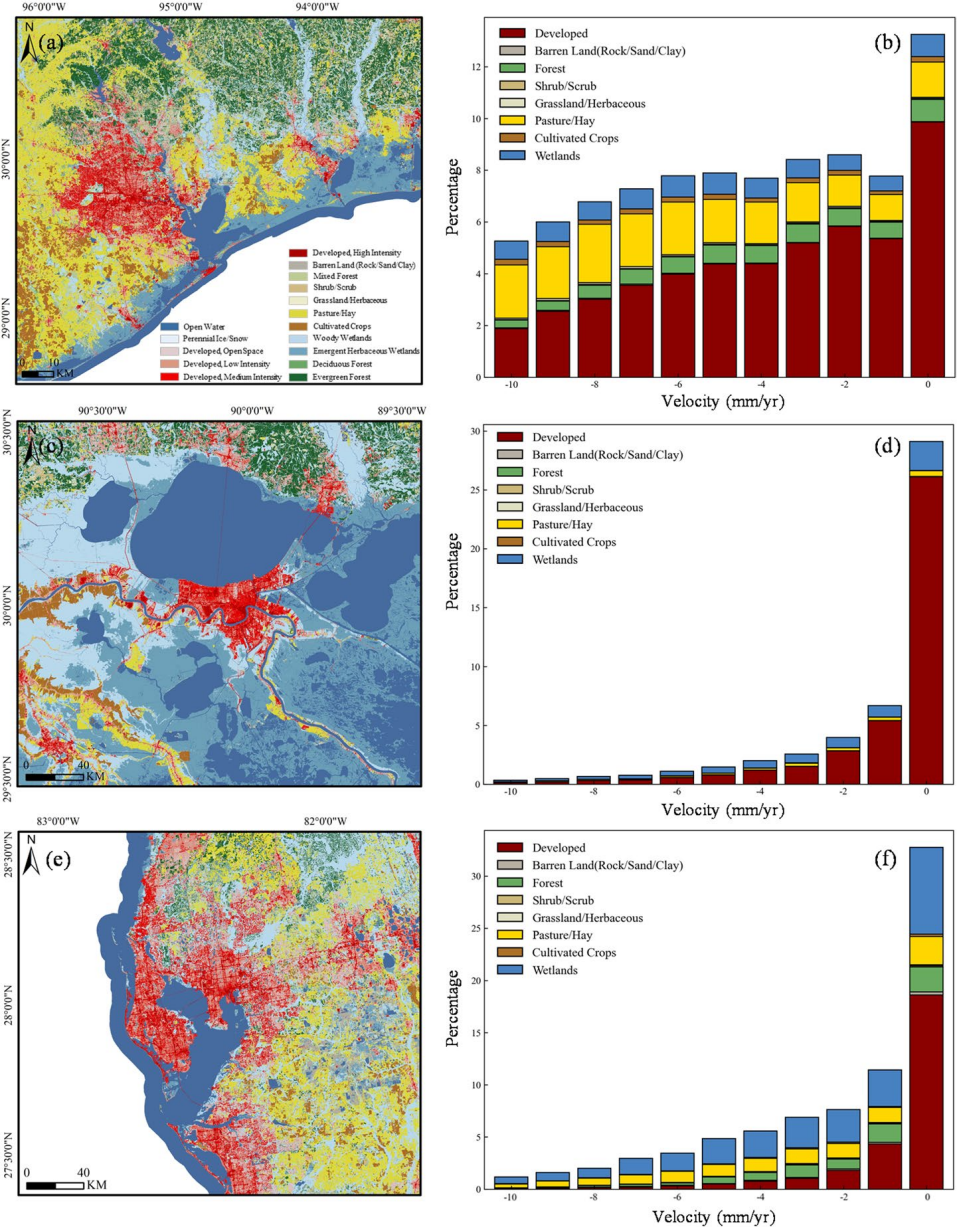
Exposure of land cover to subsidence based on the USGS 2023 NLC map: **a** NLC map for Houston; **b** Subsidence frequency distribution for different NLC in Houston; **c** NLC map for New Orleans; **d** Subsidence frequency distribution for different NLC in New Orleans; **e** NLC map for Tampa; **f** Subsidence frequency distribution for different NLC in Tampa

To evaluate the exposure of different coastal systems to subsidence, we used the 2023 National Land Cover (NLC) map from the United States Geological Survey (USGS). Land deformation rates were mapped onto NLC pixels, and subsidence exposure was determined by analyzing the distribution of InSAR pixels across various land cover types (Fig. 5a, c, e).

In Houston, New Orleans, and Tampa, land subsidence exhibits distinct patterns across different land cover types, reflecting variations in urbanization, hydrology, and land use practices (Fig. 5b, d, f and Table 2), bold indicating top three subsidence velocity in these cities. In Houston, developed areas (60.52%) and pasture/hay lands (19.86%) are most affected, with average subsidence velocities of 3.86 mm/year and 9.61 mm/year, respectively, followed by wetlands (8.19 mm/year) and forests (5.07 mm/year). New Orleans is dominated by developed land cover (84.57%), with a relatively low deformation velocity of 0.74 mm/year. However, wetlands (12.04%) and pasture/hay lands (2.70%) experience higher subsidence velocities (0.90 mm/year and 2.05 mm/year, respectively). Tampa shows a different pattern, with wetlands (21.00%) and forests (14.19%) being the most exposed land cover types, exhibiting subsidence velocities of 1.12 mm/year and 0.43 mm/year, respectively. Developed areas (42.74%) show minimal subsidence (0.06 mm/year), reflecting less pronounced urban impacts compared to Houston or New Orleans.

**Table 2:** Exposure of NLC to land subsidence

	Houston		New Orleans		Tampa	
	% Exposure	Vel (mm/year)	% Exposure	Vel (mm/year)	% Exposure	Vel (mm/year)
Developed	60.52	− 3.86	84.57	0.74	42.74	0.06
Barren Land	0.33	**− 10.14**	0.03	**− 11.00**	1.97	− 0.63
Forest	7.29	− 5.07	0.27	1.10	14.19	− 0.43
Shrub/Scrub	0.36	− 5.54	0.00	0	0.68	− 0.74
Grassland/Herbaceous	0.63	− 7.26	0.03	**− 12.05**	0.68	1.10
Pasture/Hay	19.86	**− 9.61**	2.70	− 2.05	18.13	**− 2.63**
Cultivated Crops	2.30	**− 11.46**	0.36	**− 2.40**	1.28	**− 2.24**
Wetlands	8.71	− 8.19	12.04	− 0.90	21.00	**− 1.12**

**Fig. 6:**
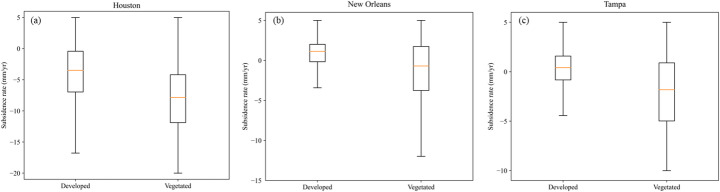
Comparison of subsidence rates between developed and vegetated areas

To further evaluate whether subsidence differs systematically across land-cover types, we compared subsidence rates between developed and vegetated areas using Mann–Whitney U tests for each city. The Mann–Whitney U test is a non-parametric statistical test used to compare two independent samples and determine whether there is a significant difference between the two data groups Unlike the traditional t-test, the Mann–Whitney U test does not require the data to follow a normal distribution (Saad et al. 2024), therefore, it is very suitable for the subsidence data. The results show that vegetated areas exhibit significantly higher subsidence rates than developed regions in all three study areas (all *p* < 0.001) as shown in Fig. 6. In Houston, the mean subsidence rate is − 8.18 mm/year in vegetated areas and − 3.94 mm/year in developed areas, with median values of − 7.85 and − 3.48 mm/year, respectively. In New Orleans, the corresponding mean rates are − 1.29 mm/year and 0.74 mm/year, and the median values are − 0.69 mm/year and 1.13 mm/year. In Tampa, the mean rates are − 2.16 mm/year and 0.21 mm/year, with median values of − 1.82 mm/year and 0.42 mm/year, respectively.

### Land subsidence caused by groundwater withdrawal

#### Houston

**Fig. 7:**
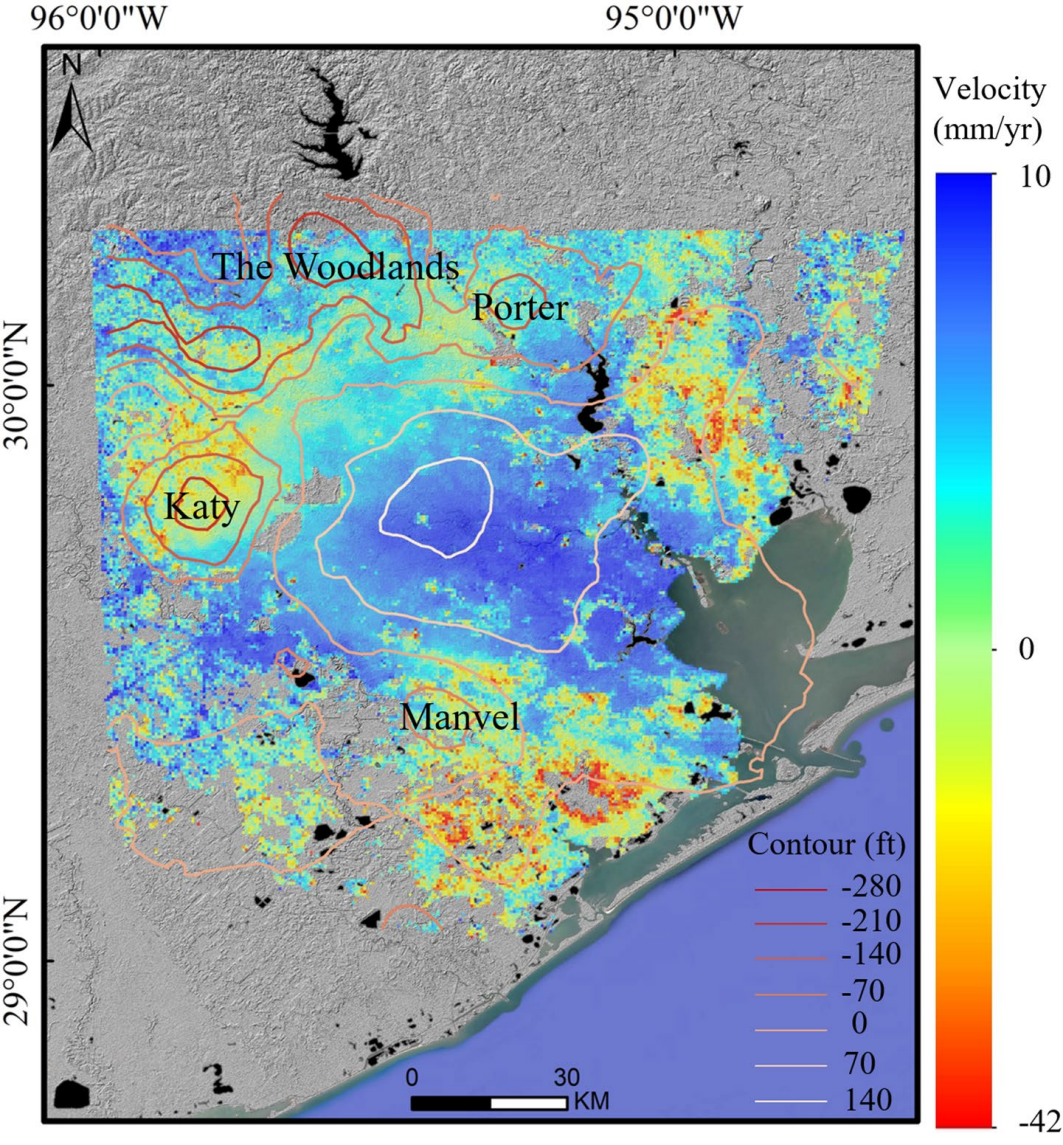
Contours of Groundwater Level Changes in Houston (1977–2024)

Several studies have known and supported the relationship between groundwater withdrawal and subsidence (Cigna and Tapete 2021; Su et al. 2021; Fiaschi and Wdowinski 2020). In Houston, subsidence has been observed since the early twentieth century, largely driven by excessive groundwater extraction from local aquifers. In Houston, for instance, significant water level declines have led to subsidence of nearly three meters by 1979. To quantify this relationship, InSAR-derived deformation rates are analyzed alongside groundwater level variations, revealing spatial correlations between subsidence patterns and groundwater depletion across the study area (Fig. 7).

From Fig. 7, we can see that there are strong correlations between the land subsidence and groundwater level changes, where the water level decline typically lowers the ground surface (Qu et al. 2023). Four distinct declining bowls, characterized by significant groundwater depletion, are located in Manvel, Katy, The Woodlands, and Porter. In these regions, subsidence rates exceed 10 mm/year, reflecting the significant impact of groundwater extraction on land stability. In contrast, areas where groundwater levels remain more stable or are rising, such as parts of central Houston, show lower subsidence rates or even slight uplift. As subsidence exhibits clear spatial variability, the relationships between deformation and controlling factors are not consistent across the study area. Under such conditions, the GWR model is applied to account for spatial variability and examine how the influence of subsurface factors varies across locations. In Houston, the model shows a strong fit, with an R^2^ value of 0.645, indicating that groundwater-related factors explain a large portion of the observed deformation. The mean coefficient for groundwater change is 0.215, indicating a strong overall association between groundwater decline and surface deformation. Notably, the coefficients exhibit a wide range, varying from − 1.844 to 2.549, which reflects substantial spatial heterogeneity in the groundwater subsidence relationship. While groundwater decline generally contributes to subsidence, its influence differs significantly across locations, likely due to variations in local hydrogeological conditions and soil properties.

#### New Orleans

Groundwater withdrawal has long been recognized as a major cause of land subsidence in industrial areas (Jones et al. 2016). In the Eastern region of New Orleans, there are two main industrial areas, including Taft and Norco. Our result reveals that the subsidence patterns in the industrial areas between 2019 and 2024 are localized, particularly near Diamond Green Diesel in Norco, where subsidence rates reached approximately 10 mm/year. In New Orleans, the GWR model shows a moderate fit, with an R^2^ value of approximately 0.33, indicating that groundwater-related factors explain part of the observed deformation but are not the sole controlling mechanism. The local coefficients for groundwater-related factors range from − 3.507 to 6.194, with a mean value of 0.263. This wide range indicates substantial variation in both magnitude and direction across the study area, reflecting strong spatial heterogeneity in the groundwater–subsidence relationship.

**Fig. 8:**
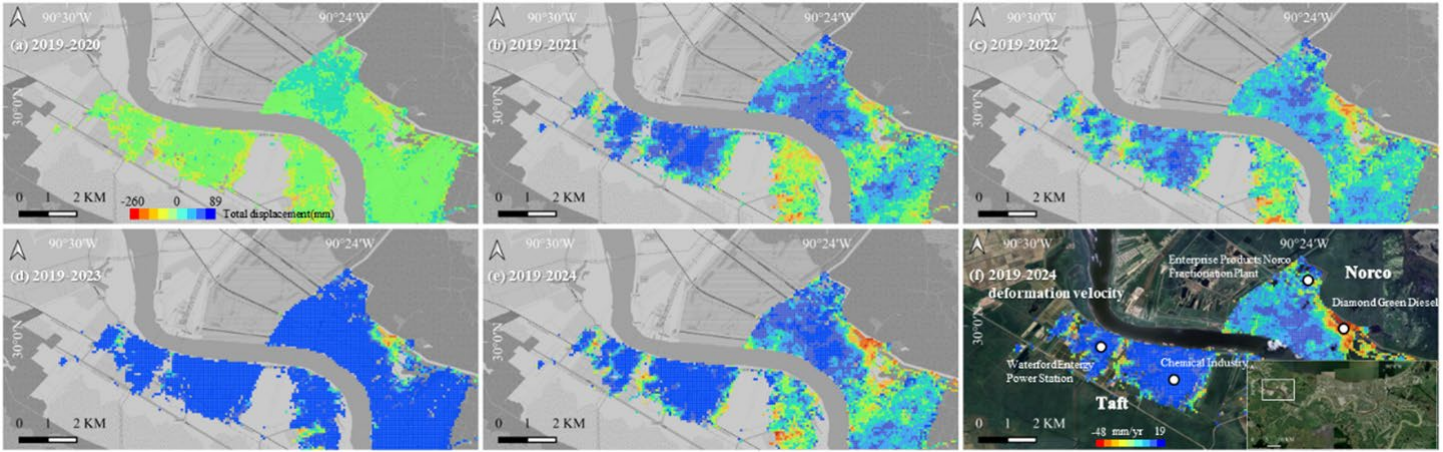
Deformation evolution in Taft and Norco: **a** 2019–2020; **b** 2019–2021; **c** 2019–2022; **d** 2019–2023; **e** 2019–2024; **f** Average deformation rate in the area

To study the deformation evolution process, the SAR image of January 12, 2019, was used as a reference to obtain the deformation evolution process, and the results are shown in Fig. 8. The results indicate that significant subsidence was not observed in the initial years; however, in the latter two years, it became more persistent and expanded toward industrial facilities, including the chemical industries and power stations. Notably, an uplift event was observed in 2023, followed by renewed subsidence, likely linked to industrial activities, including groundwater extraction, that remain active (Dokka 2011; Jones et al. 2016; Qu et al. 2023).

**Fig. 9:**
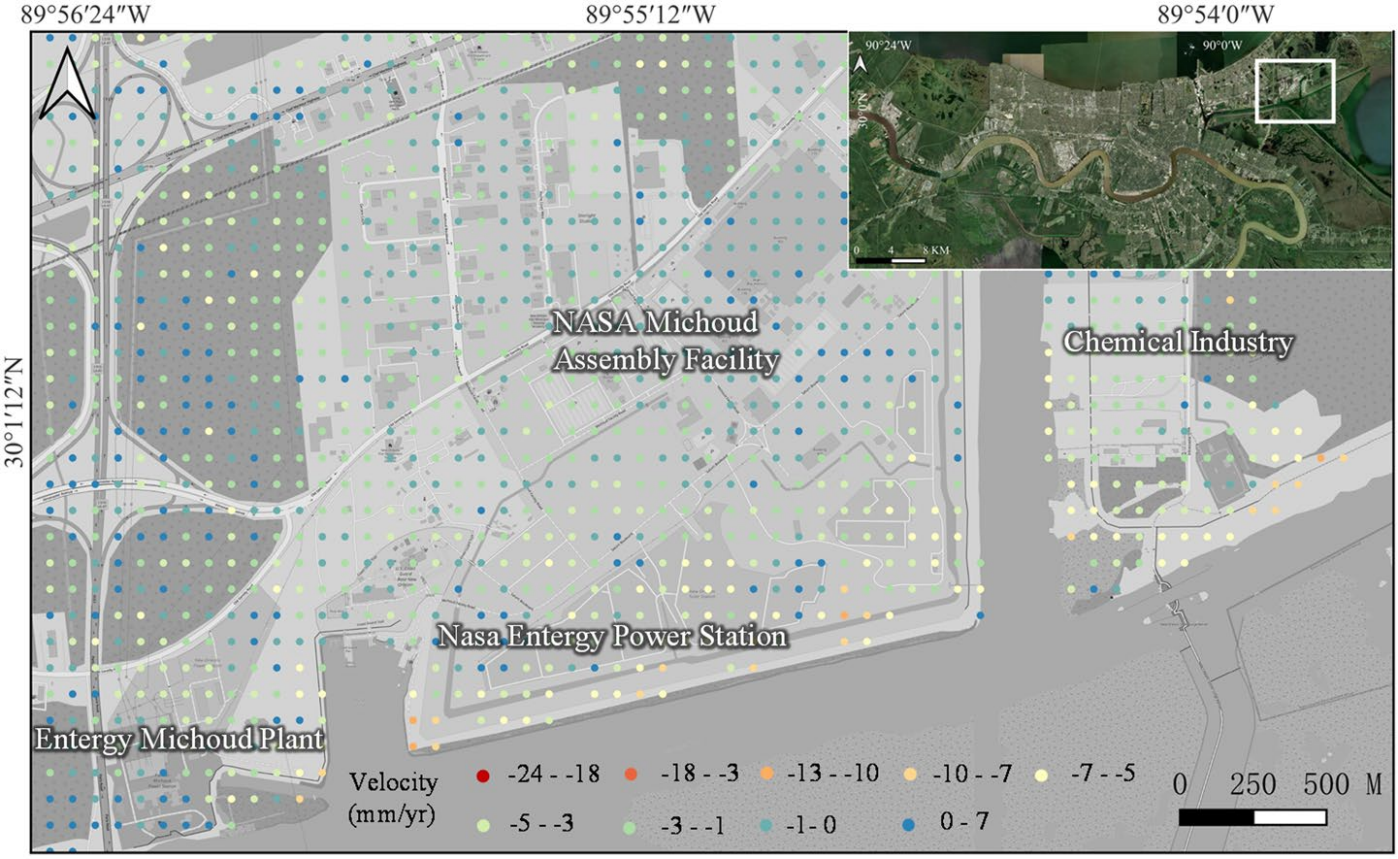
Subsidence in Michoud

Figure 9 provides a detailed view of subsidence rates in the Michoud area, which has also experienced ground deformation. Previous studies showed that significant subsidence occurred in this region, with rates of 25–30 mm/year, primarily attributed to groundwater extraction for power generation, as indicated by UAVSAR data from 2009 to 2012 (Jones et al. 2016). Following the closure of the power station in 2016 and the cessation of groundwater extraction, our results indicate a substantial reduction in the subsidence rate to 5–7 mm/year. These findings suggest that halting groundwater extraction can effectively mitigate subsidence. However, much of the subsidence caused by groundwater withdrawal is often irreversible, as soil compaction and structural changes in the subsurface prevent full recovery. As a result, slight subsidence continues in the area.

#### Tampa

**Fig. 10:**
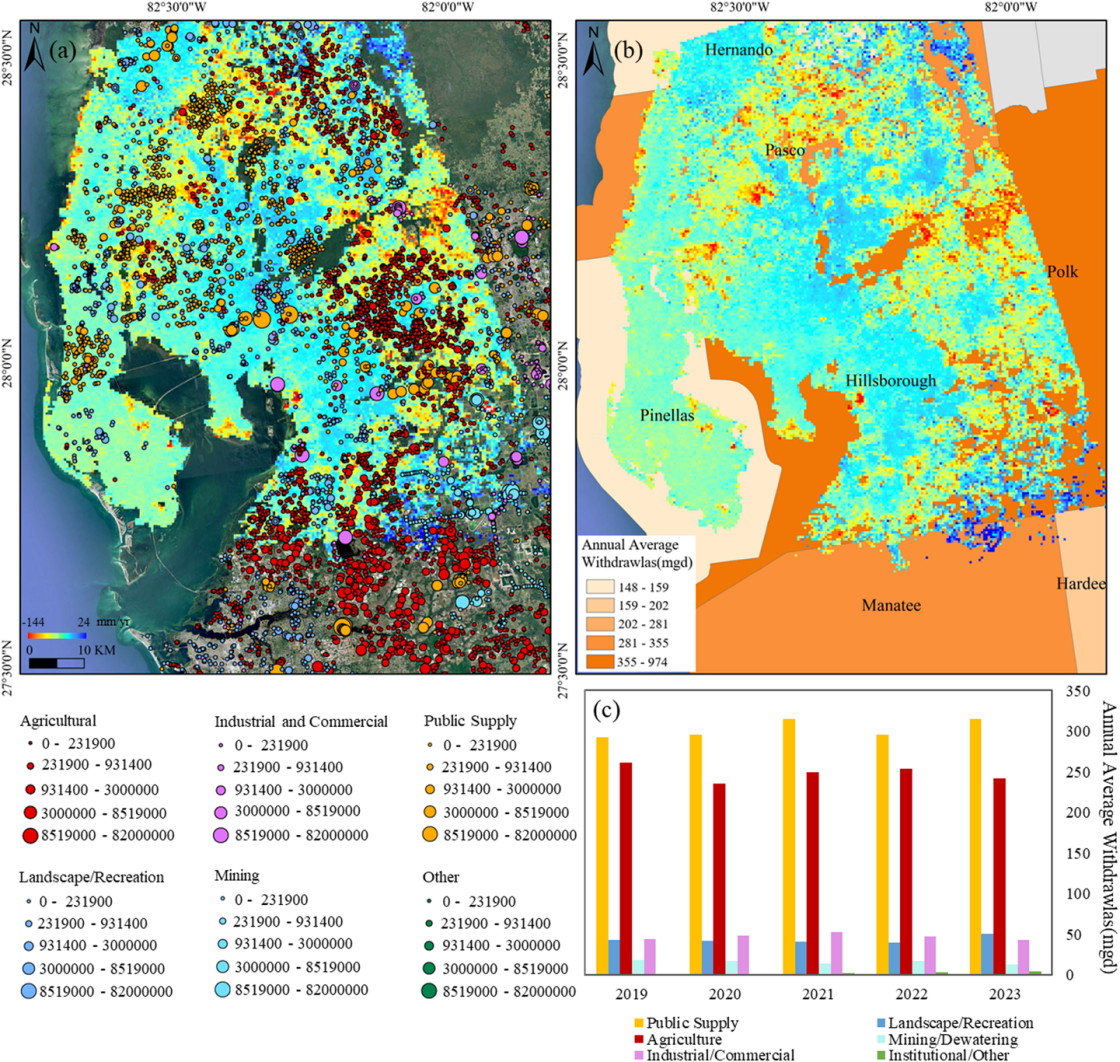
Water use in Tampa: **a** Well Use Permits Distribution; **b** Annual water withdrawals across the study area; **c** Trends in Water Withdrawals by Use Type (2019–2023)

Figure 10 shows the relationship between groundwater use and land subsidence in the Tampa area. The well use permits map (Fig. 10a) shows a dense concentration of groundwater wells, particularly in the eastern and southern regions, where agricultural and industrial activities are dominant. These areas correspond closely to the high-subsidence zones, indicated by red and orange colors. Significant subsidence is also observed in the northern region, particularly in Pasco County, where groundwater withdrawals for public supply are substantial. The annual groundwater withdrawal map (Fig. 10b) highlights the intensity of water use across different counties. Polk and Hillsborough counties, shown in darker orange, record the highest withdrawals (355–974 Mgal/d), coinciding with areas of significant land subsidence. This pattern suggests that prolonged groundwater extraction is driving aquifer compaction, leading to measurable ground deformation. In comparison, Pinellas and Hernando counties, with lower withdrawals (148–159 Mgal/d), exhibit minimal subsidence.

Over the period from 2019 to 2023 (Fig. 10c), total groundwater withdrawals averaged 660.71 Mgal/d, with public supply and agriculture as the primary consumers. While public supply remained stable, agricultural withdrawals fluctuated, likely due to varying climatic conditions. Industrial and commercial use peaked in 2021 before declining, whereas landscape and recreational water use showed steady growth, particularly in 2023. Mining and dewatering withdrawals continued to decline throughout the period.

**Fig. 11:**
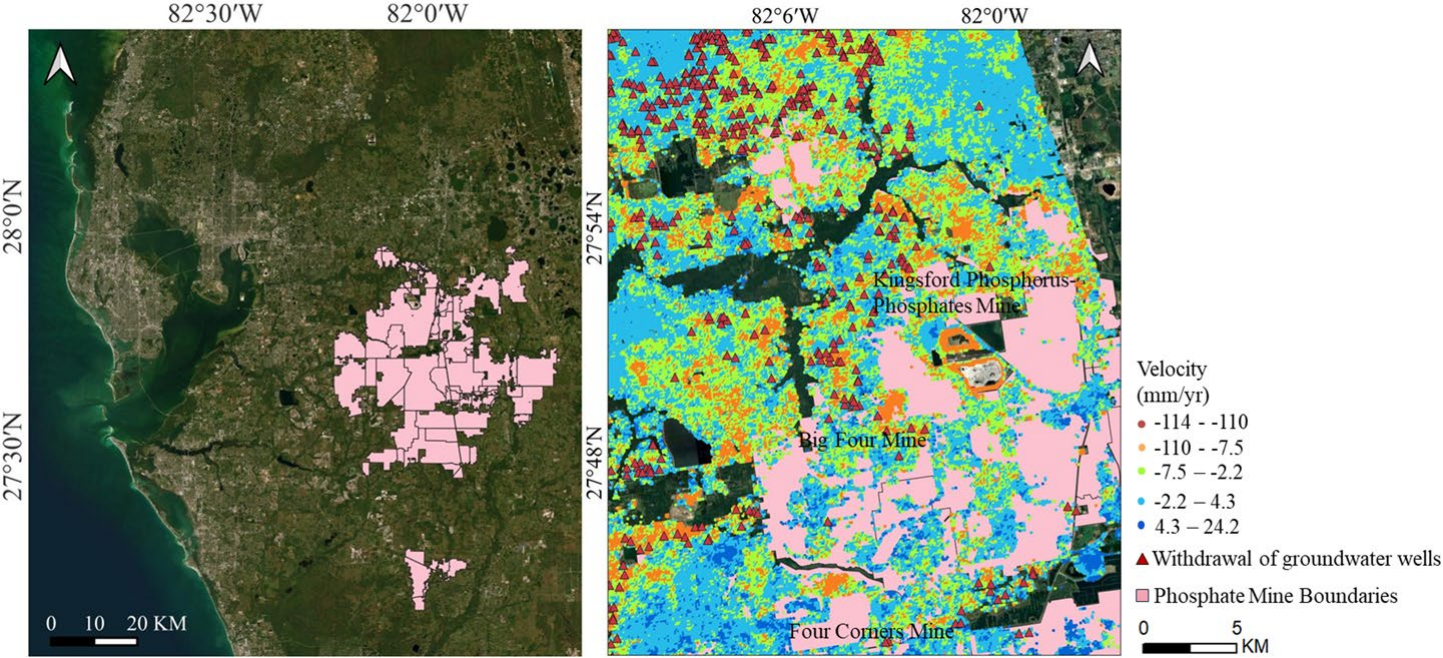
Spatial distribution of subsidence rates and phosphate mining

Additionally, mining activities also contributed to subsidence. Currently, Florida hosts 27 phosphate mines spanning over 1820 km^2^, accounting for approximately 1% of the state’s total land area (Florida Department of Environmental Protection 2013). Our study identifies significant subsidence in mining areas at a rate of 7 mm/year (Fig. 11). The most severe subsidence in Tampa occurs at Kingsford Phosphorus-Phosphates Mine, with rates ranging from 25 to 110 mm/year. Four Corners Mine also remains active, experiencing subsidence at 28 mm/year. Other mining sites, such as Big Four Mine, exhibit slight subsidence, with rates reaching up to 10 mm/year.

Land subsidence in mines is generally the result of the combined effects of mining disturbance forces and gravity. The rock mass in the mining area moves, deforms, or even is destroyed due to mining disturbance, and then the overlying soft sediments are compressed and deformed under the action of gravity, eventually leading to significant surface subsidence (Sainsbury 2012; Wardle and Enever 1983). Additionally, dewatering processes commonly associated with mining operations, particularly in open-pit and underground mines, lower the groundwater table. This reduction in hydraulic pressure can lead to soil consolidation, further contributing to subsidence (Fathi Salmi et al. 2017; Reddish and Whittaker 2012). Analysis of well data suggests a strong correlation between subsidence in mining areas and groundwater extraction. In regions with notable subsidence (10–40 mm/year), a high concentration of wells used for dewatering or water supply is observed. Furthermore, the proximity of these wells to mining sites suggests a combined effect of resource extraction and groundwater pumping on ground stability. In contrast, mining areas with minimal subsidence are typically surrounded by wells used for monitoring or surface water extraction, implying a reduced impact on the groundwater system. The GWR model yields an R^2^ value of 0.5, indicating a moderate to relatively strong explanatory capacity of groundwater-related factors for the observed deformation patterns. In addition, in the mining region, the distribution of well density within a 2 km radius aligns with areas of increased subsidence, suggesting that localized groundwater extraction contributes to deformation in these zones.

### Land subsidence caused by hydrocarbon exploration

#### Houston

**Fig. 12:**
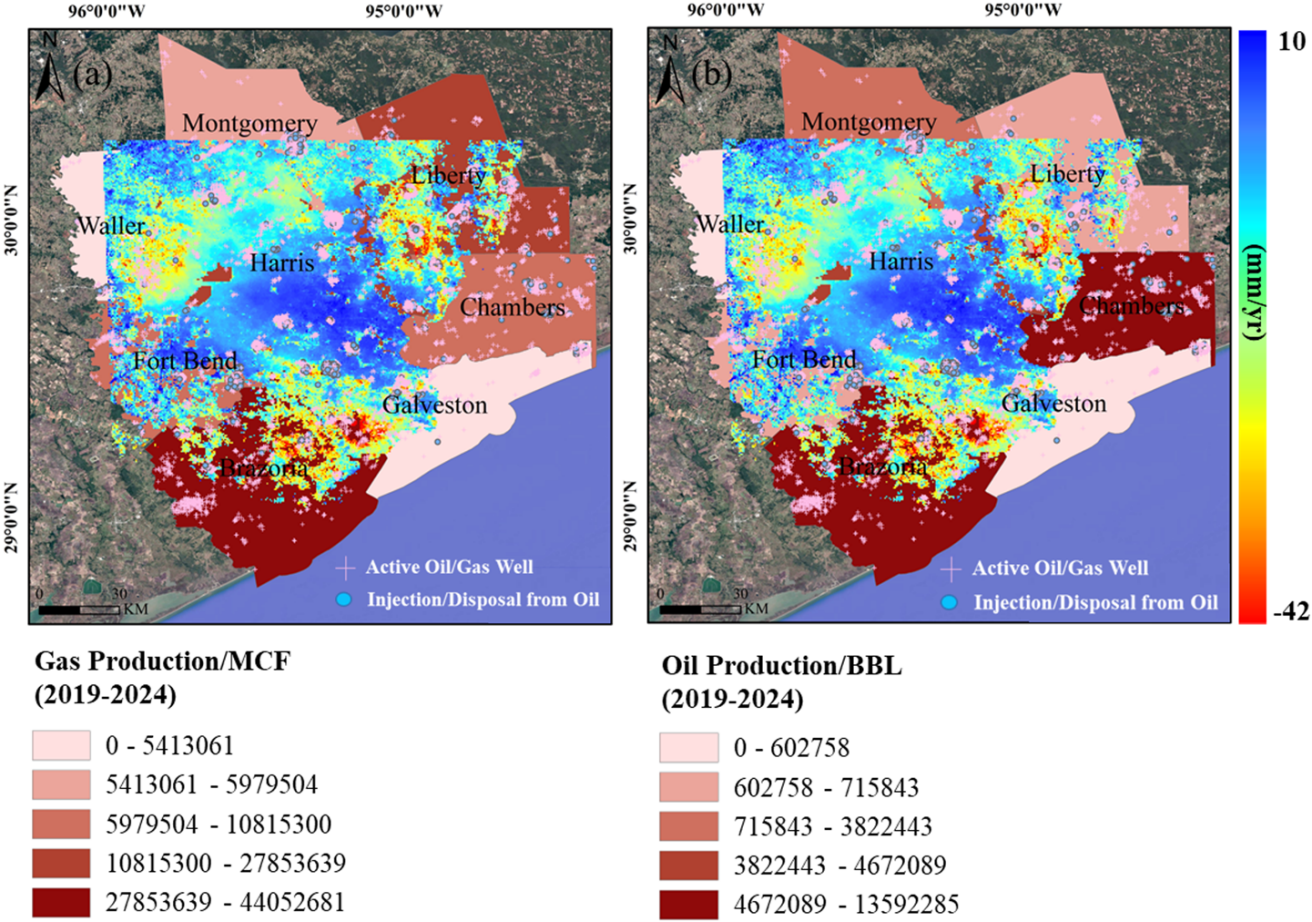
Oil (**a**) and gas (**b**) production by county overlaid with InSAR-derived deformation rates

Land subsidence closely correlates with the density of oil and gas wells, with the most pronounced subsidence occurring in regions of highest well concentration. As shown in Fig. 12, active wells, marked by pink cross symbols, are predominantly clustered in areas with significant subsidence, highlighted in red on the map. This spatial correlation suggests that extensive hydrocarbon extraction plays a key role in surface deformation. The concentration of active wells likely leads to substantial reservoir pressure depletion, a well-documented cause of subsidence. As hydrocarbons are extracted, the resulting reduction in pore pressure causes the geological formations to compact, leading to land subsidence.

The examination of subsidence patterns in conjunction with oil and gas production data from 2019 to 2024 reveals a notable correlation between subsidence severity and extraction activities across several counties. Three regions, Chambers, Liberty, and Brazoria counties, show significant subsidence, each with distinct deformation patterns. In these areas, dense clusters of active wells and extensive extraction intensify subsidence. Brazoria County is the most affected, with subsidence rates ranging from 15 to 35 mm/year, followed by Liberty County at 10 to 25 mm/year. Waller County, with rates between 10 and 20 mm/year, experiences the least deformation, due to lower well density and reduced production. Nevertheless, ongoing extraction, even at smaller scales, continues to contribute to surface deformation. The GWR model indicates that the overall explanatory impact of hydrocarbon activity is relatively limited compared with groundwater-related processes, with a mean local coefficient of 0.063 (median = 0.057). In addition, the coefficients range from − 0.399 to 0.521, reflecting spatial variability in the direction and magnitude of this relationship. These results suggest that hydrocarbon extraction contributes to subsidence in Houston, but its impact is secondary.

#### New Orleans

Groundwater extraction is a major cause of land subsidence in the southern region, particularly around the Mississippi Delta. However, oil and gas exploration and production activities also contribute to subsidence in this region. South of Lake Pontchartrain, numerous oil and gas fields, such as those in the Mississippi Delta, have been actively explored since the late 1950s, and their extraction activities have been shown to influence land subsidence (Kolker et al. 2011). The GWR model gives an R^2^ of 0.307. The mean coefficient is 0.123; therefore, the overall association with subsidence is positive but not particularly strong. The coefficients range from − 1.278 to 1.014, which shows that the relationship changes across space, although the variation is smaller than that seen for groundwater. Hydrocarbon extraction appears to be related to subsidence in some areas, but the effect is not consistent and tends to be limited.

**Fig. 13:**
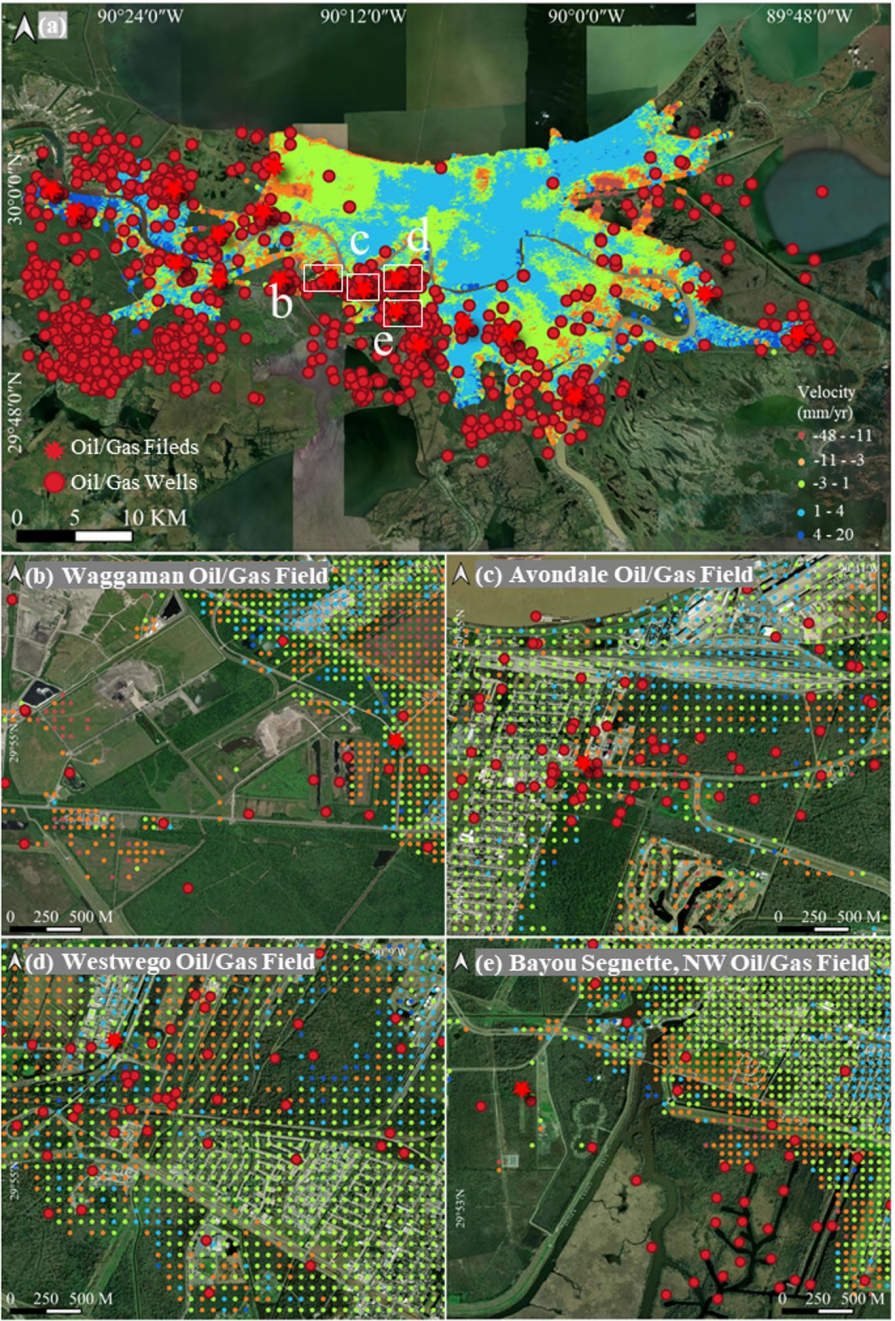
**a** Subsidence in New Orleans with oil/gas fields and wells distribution; **b**–**e** Close-up views at Waggaman field, Avondale field, Westwego field, and Bayou Segnette field separately

Figure 13a illustrates that ground deformation is notably evident around oil and gas fields. To examine the impact of extraction activities on land subsidence, we analyzed four fields in the southern region. The InSAR results reveal localized subsidence rates of 5–15 mm/year near the Avondale field, 5–10 mm/year near the Westwego field, 10–30 mm/year at the Waggaman field, and 5–20 mm/year at the Bayou Segnett-NW field (Fig. 13b–e). These areas are characterized by a dense concentration of active wells, and their subsidence rates during production are significantly higher than those in surrounding areas. However, the resulting deformation extends beyond the field boundaries, affecting a broader area (Gambolati and Teatini 2015). The results indicate that the influence of oil and gas extraction on subsidence is not limited to the production zone but also impacts neighboring fields or zones within the same field.

**Fig. 14:**
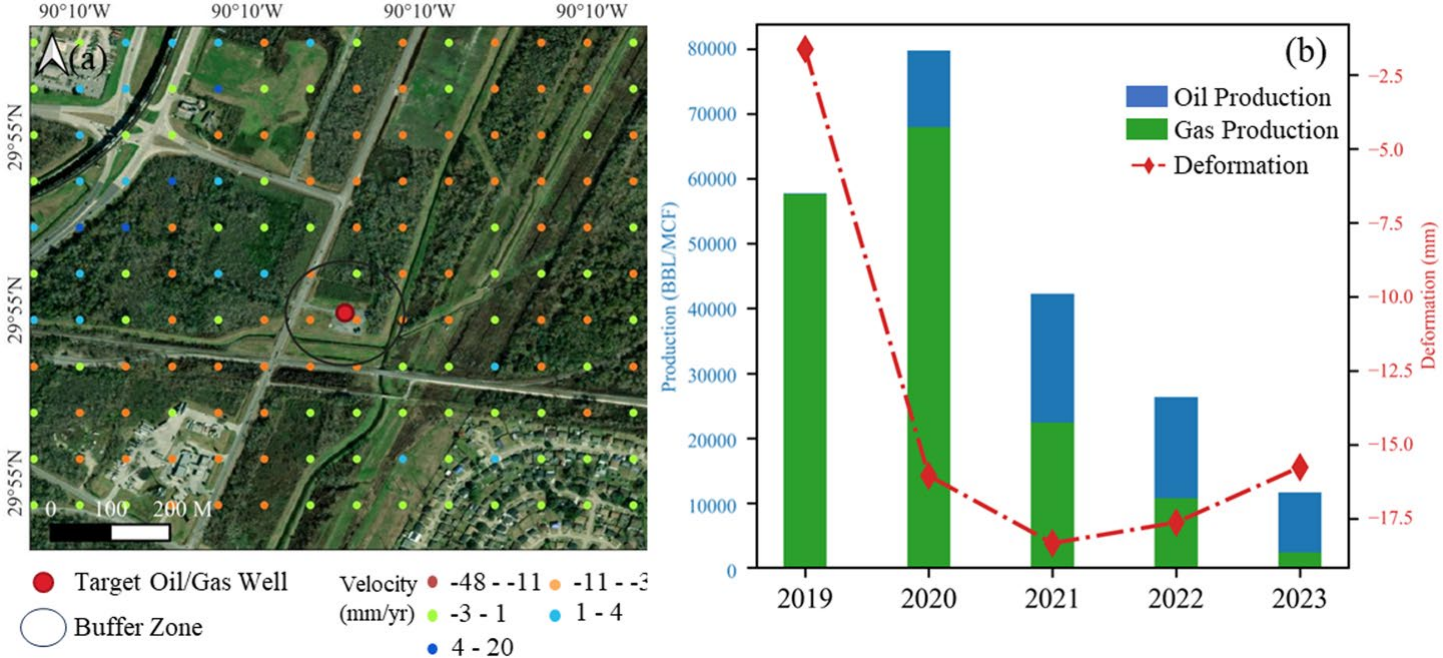
**a** Selected well with a 1 km buffer zone and InSAR deformation velocity; **b** Stacked oil and gas production data alongside deformation measurements

We select a particularly active well from the Avondale oil field and average the subsidence rates within a 1 km radius of the well, as shown in Fig. 14. From 1977 to 2014, the total oil production from these fields amounted to 12,195,052 barrels, and gas production reached 3,896,245 barrels. The ground deformation shows significant variation, particularly in 2020 when there was a sharp increase in both oil and gas production, coinciding with a marked negative deformation value (− 16.04 mm). Despite a decrease in both oil and gas production in subsequent years, the region remains subsiding, suggesting that subsidence continues, possibly linked to the creation of underground voids or changes in the strata due to extraction activities.

### Land subsidence caused by shallow sediment compaction

In New Orleans, the urban expansion occurred on softer, swampy soils further from the river (Asselen et al. 2024). In these low-lying areas, historical drainage and land development have resulted in land subsidence, primarily due to the oxidation and degradation of peat soils and compaction of soft organic materials. As groundwater levels drop, shallow peat is exposed to air, initiating oxidation and further contributing to land subsidence (Asselen et al. 2019).

Our results indicate that subsidence has occurred in several areas within the New Orleans Metropolitan area, including Lakeview, Milneburg, and the Desire Area (Fig. 3a). These regions, which are known to have high subsidence vulnerability, coincide with areas identified in the 2019 report. The subsidence in these areas is primarily due to the presence of shallow peat and organic soils, which undergo oxidation and compaction when exposed to air as groundwater levels decline.

**Fig. 15:**
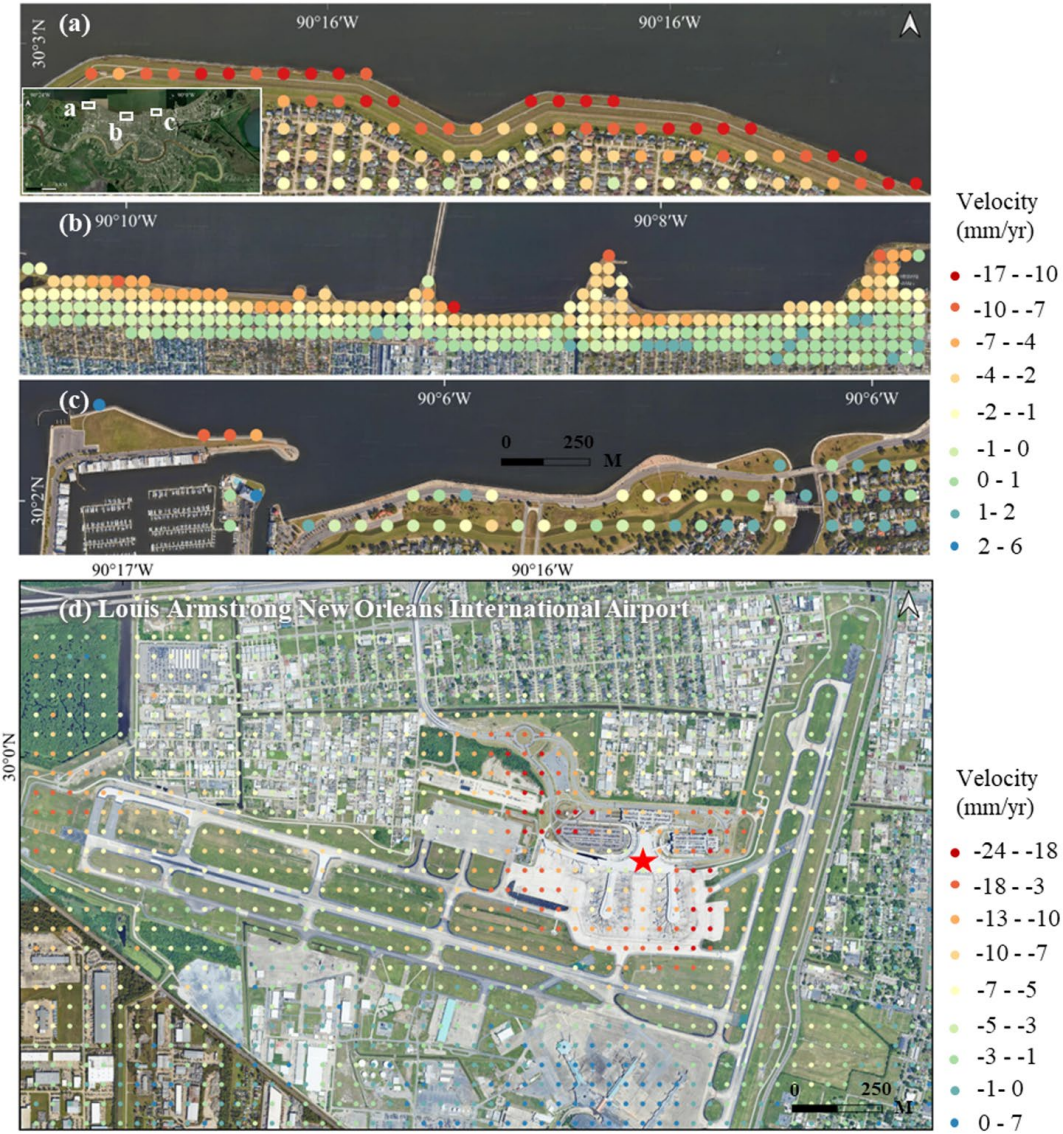
Subsidence in **a**–**c** coastal levees and **d** airport areas

The areas bordering Lake Pontchartrain and the Mississippi River have experienced significant subsidence at an average rate of 10 mm/year. As shown in Fig. 15a–c, the most pronounced subsidence occurs near the coastline and within the levee system. Although the levee system is critical for protecting lives and property from flooding, it disrupts the natural flow of river water into the wetlands. This disruption reduces the delivery of fresh sediment, which is essential for sustaining wetland vegetation and promoting land accretion. Consequently, the coastal marshes are also experiencing significant subsidence.

New Orleans Airport has also experienced ongoing subsidence (Fig. 15d) driven by geological factors. The airport is situated in the Mississippi River Delta, where the underlying geology is composed of soft clay, peat, and sedimentary deposits. These materials are highly compressible, making the area prone to natural consolidation and subsidence over time. The construction of airport infrastructure, such as runways, taxiways, and terminals, has further intensified the compression of these soft soils. Moreover, the natural settling of deltaic sediments, a long-standing process in the region, has contributed to the gradual sinking of the ground. Together, these factors have led to the persistent subsidence observed at the airport.

**Fig. 16:**
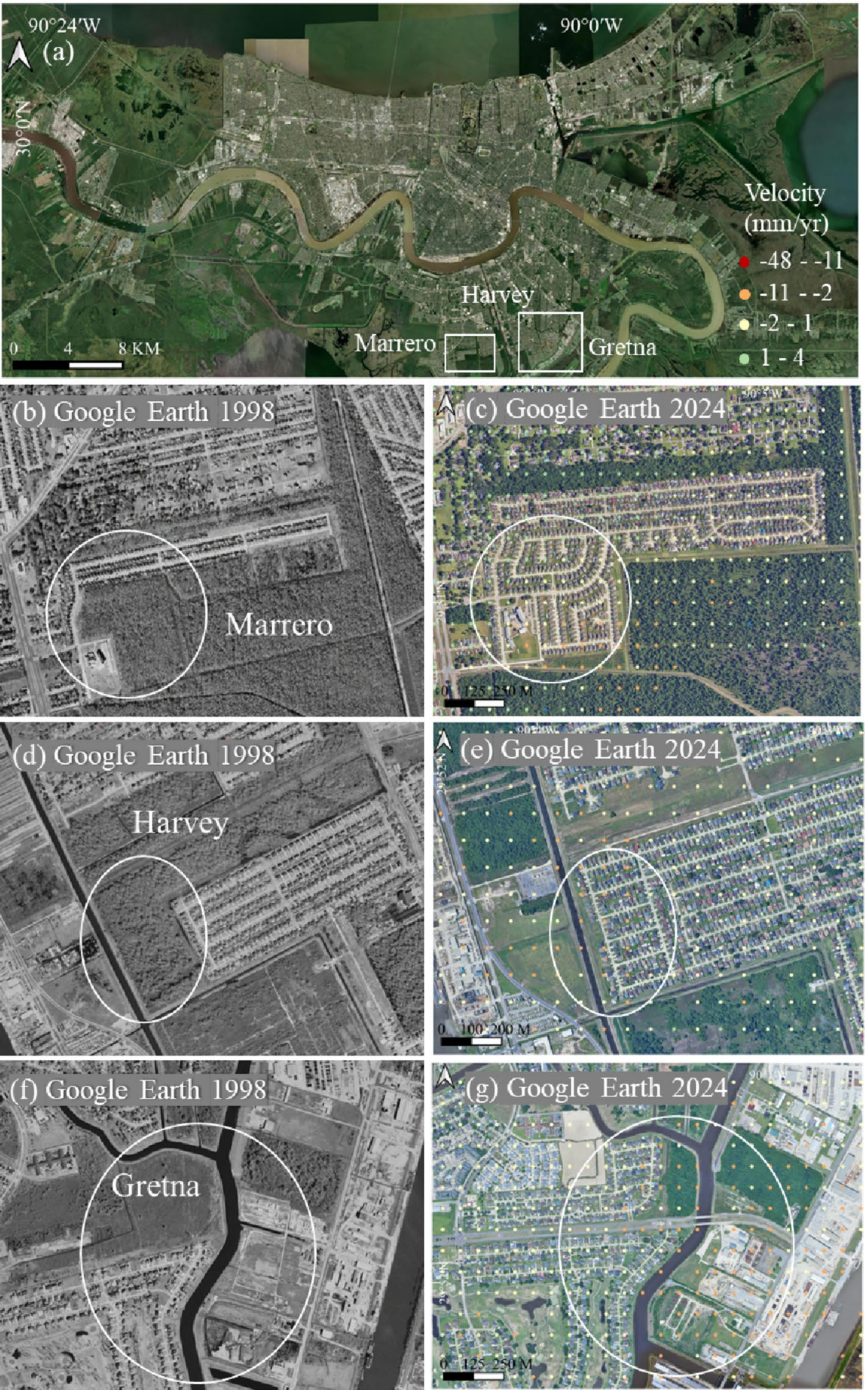
Subsidence in three neighborhoods of southern New Orleans, with locations shown in (**a**); **b**–**g** display InSAR deformation velocities overlaid on the 1998 and 2024 optical images

Figure 16 illustrates multiple neighborhoods in Marrero, Harvey, and Gretna that were developed before 1998. In these newly constructed blocks, which were built on former wetlands, subsidence is more pronounced compared to the surrounding structures.

The predominant soils in these areas are Schriever and Westwego clay. Schriever, characterized by high compressibility and low permeability, undergoes rapid shallow compaction under applied loads, with settlement often occurring within the first few years of construction. Additionally, Barbary muck, rich in organic matter, contributes to long-term subsidence through organic decomposition when exposed to drainage or oxygenation. These processes, compounded by potential groundwater fluctuations, result in both rapid initial settlement and prolonged subsidence. The soils of Westwego formed from semifluid clayey alluvium and organic material, which underwent irreversible shrinkage in the upper layers due to artificial drainage. This shallow compaction likely contributes to a substantial portion of the observed subsidence, yet it often goes undetected by benchmarks or CORS GPS stations. These measurement points are typically installed on commercial buildings or foundations that are anchored in the more stable Pleistocene layer, making them less sensitive to surface-level settlement processes.

## Future flooding hazards

Future inundation was estimated by combining projected sea level rise with InSAR-derived land subsidence using a static, elevation-based approach. Our main goal is to identify areas that may become more exposed under combined sea-level rise and subsidence, rather than to produce detailed or event-specific flood predictions. Within this framework, processes such as hydrodynamic interactions, storm surge dynamics, and infrastructure performance (e.g., levees and drainage systems) are not explicitly represented, and the results should therefore be interpreted as indicative estimates of potential inundation rather than precise flood predictions. Similar approaches have been used in previous studies to provide large-scale estimates of coastal inundation (e.g., Dada et al. 2023; Kanan et al. 2023; Gao et al. 2021; Kirezci et al. 2020; Sande et al. 2012; Strauss et al. 2012).

To achieve this, subsidence rates are resampled onto a 30 × 30 m grid based on the SRTM DEM, providing a consistent topographic framework for regional-scale analysis. It should be noted that the use of SRTM DEM may introduce uncertainties in low-relief coastal regions due to its limited vertical accuracy. As a result, small elevation differences may influence local inundation boundaries. However, at the regional scale considered in this study, the magnitude of subsidence and projected sea-level rise is substantially larger than the vertical uncertainty of the SRTM DEM, and therefore, the DEM is sufficient for capturing broad spatial patterns of flood exposure.

**Fig. 17:**
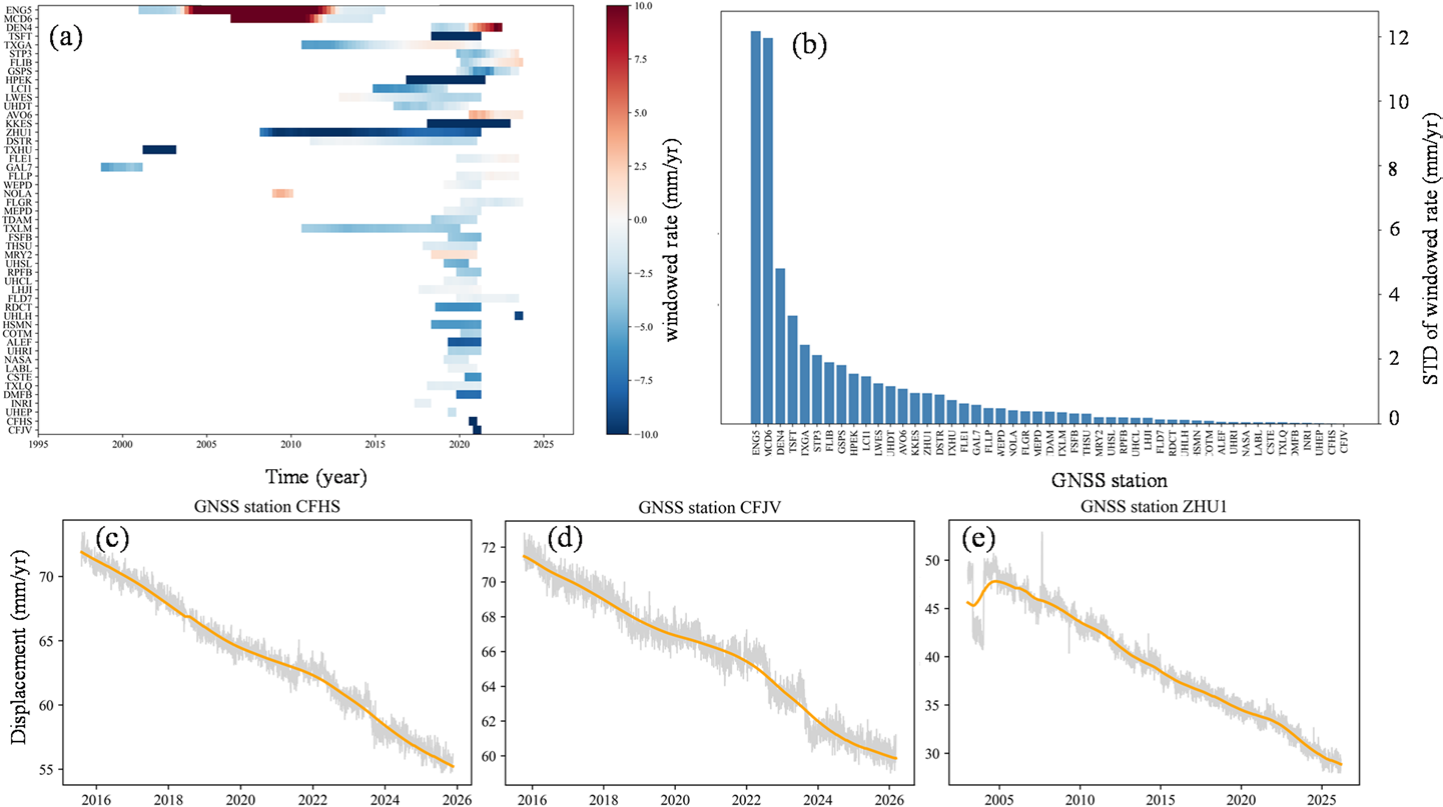
**a** Temporal evolution of deformation rates at each GNSS station derived from time series displacement using a moving-window regression approach. **b** Variability of the windowed deformation rates at each station, quantified by the standard deviation, illustrates the temporal stability of displacement. **c**–**e** SSA applied to the representative GNSS displacement time series

Assuming a linear velocity trend throughout the twenty-first century (Shirzaei et al. 2021), we adjust the elevation model to incorporate projected subsidence. To evaluate the temporal stability of land subsidence and the validity of linear extrapolation, GNSS displacement time series were analyzed using both a moving-window rate approach and Singular Spectrum Analysis (SSA) (Fig. 17). Window lengths ranging from 3 to 10 years were adopted depending on data availability, and linear velocities were estimated within each window to characterize the temporal evolution of deformation rates. The variability of these rates was quantified using the standard deviation of the windowed estimates. The results indicate that, for most stations, the variability of windowed rates remains relatively small, typically within 4 mm/year (Fig. 17b), suggesting stable long-term deformation behavior.

To further characterize the underlying trends, SSA was applied to GNSS time series with sufficiently long observation periods. The low-frequency reconstructed components were extracted to represent the long-term deformation signal. Representative examples (Fig. 17c–e) show that, despite short-term fluctuations and noise, the reconstructed trends exhibit a smooth and approximately linear behavior over decadal timescales. These results suggest that subsidence in the study area is dominated by long-term, gradually evolving processes, and that linear extrapolation provides a reasonable approximation for regional-scale hazard assessment.

### Houston

**Fig. 18:**
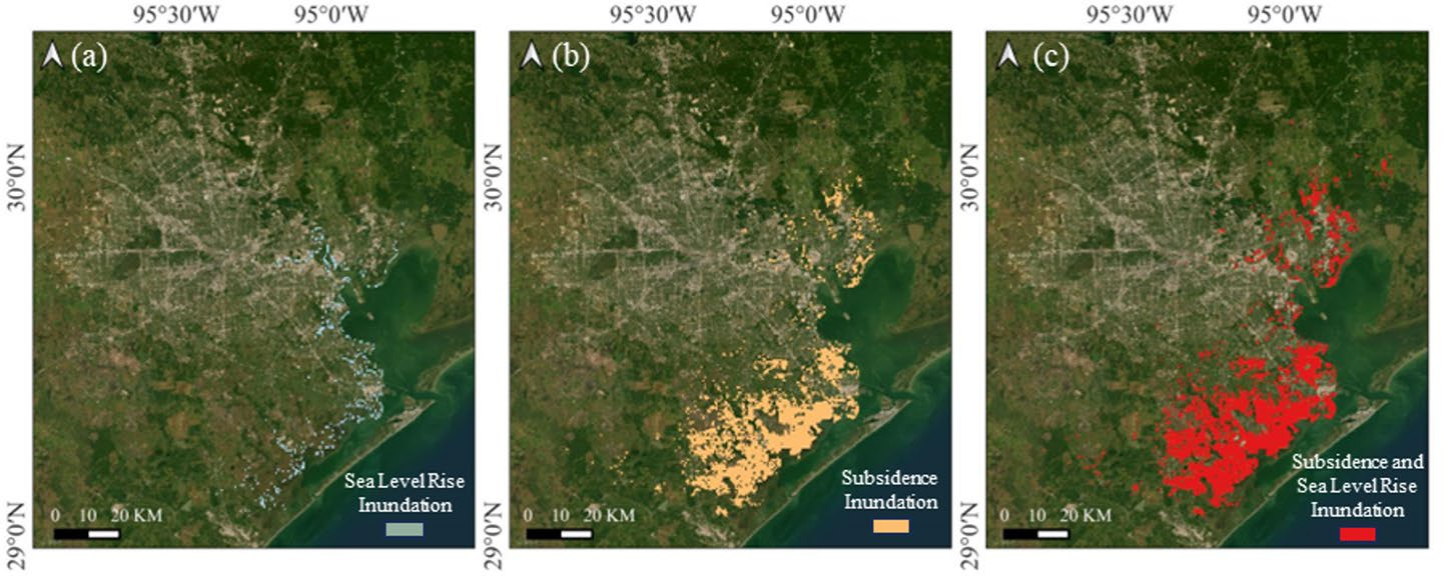
Inundation area in Houston by 2100 due to **a** sea level rise, **b** subsidence, and **c** both sea level rise and subsidence

Houston faces significant flood risks due to land subsidence and sea level rise (Fig. 18). Under the SSP5-8.5 scenario, sea levels are projected to rise by 1.29 m by 2100, leading to the inundation of approximately 124.3 km^2^ of coastal land. Low-lying areas, particularly in southeastern Houston, are at high risk as rising sea levels encroach upon marshlands and bayous that are already near or below sea level.

When land subsidence is incorporated into the analysis, the extent of inundation increases significantly, expected to impact approximately 932.6 km^2^ by 2100. Subsidence accelerates land elevation loss, reducing drainage capacity and exacerbating flooding risks. Consequently, the total affected area, when subsidence and sea level rise are considered together, expands to 1056.9 km^2^.

The combined effects of subsidence and sea level rise result in an 86% increase in total inundated area compared to the effects of sea level rise alone. This compounding effect is especially pronounced in counties such as Chambers, Liberty, Brazoria, and Galveston, where subsidence rates exceed 15 mm/year. These regions not only face direct threats from rising sea levels but also experience accelerated flood risks due to ongoing ground settlement. Without mitigation measures, these combined forces will likely lead to more frequent and severe flooding, threatening critical infrastructure, water resources, and coastal communities.

### New Orleans

**Fig. 19:**
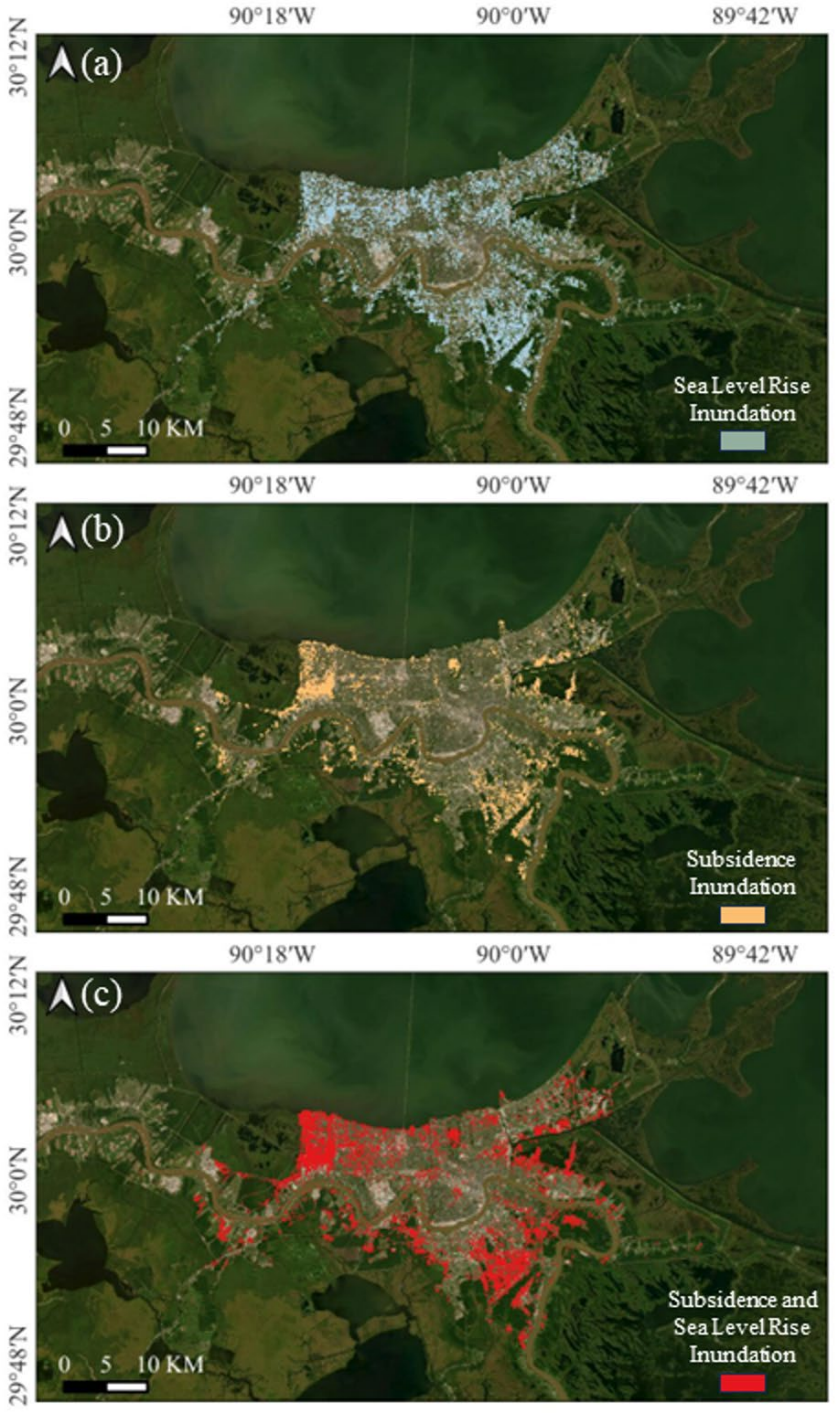
Inundation area in New Orleans by 2100 due to **a** sea level rise, **b** subsidence, and **c** both sea level rise and subsidence

Situated largely below sea level and surrounded by water, New Orleans is particularly susceptible to climate-induced changes and land deformation processes (Fig. 19). Under the SSP5-8.5 scenario, sea levels are projected to rise by 1.65 m by 2100, inundating approximately 158.1 km^2^.

Subsidence further lowers the land surface, exacerbating the impacts of rising sea levels. When both factors are considered together, the total inundated area expands to 166.5 km^2^, an increase of 8.4 km^2^ compared to considering sea level rise alone.

The most severely affected areas include Lakeview, Gentilly, Harvey, and the Lower Ninth Ward, characterized by high population densities, extensive urban infrastructure, and historical flood management challenges. Additionally, eastern New Orleans, which encompasses industrial zones and wetlands, faces significant inundation risks due to its proximity to subsiding land and water. It not only threatens residential areas but also poses serious risks to critical infrastructure, including transportation networks, levee systems, and pumping stations.

### Tampa

**Fig. 20:**
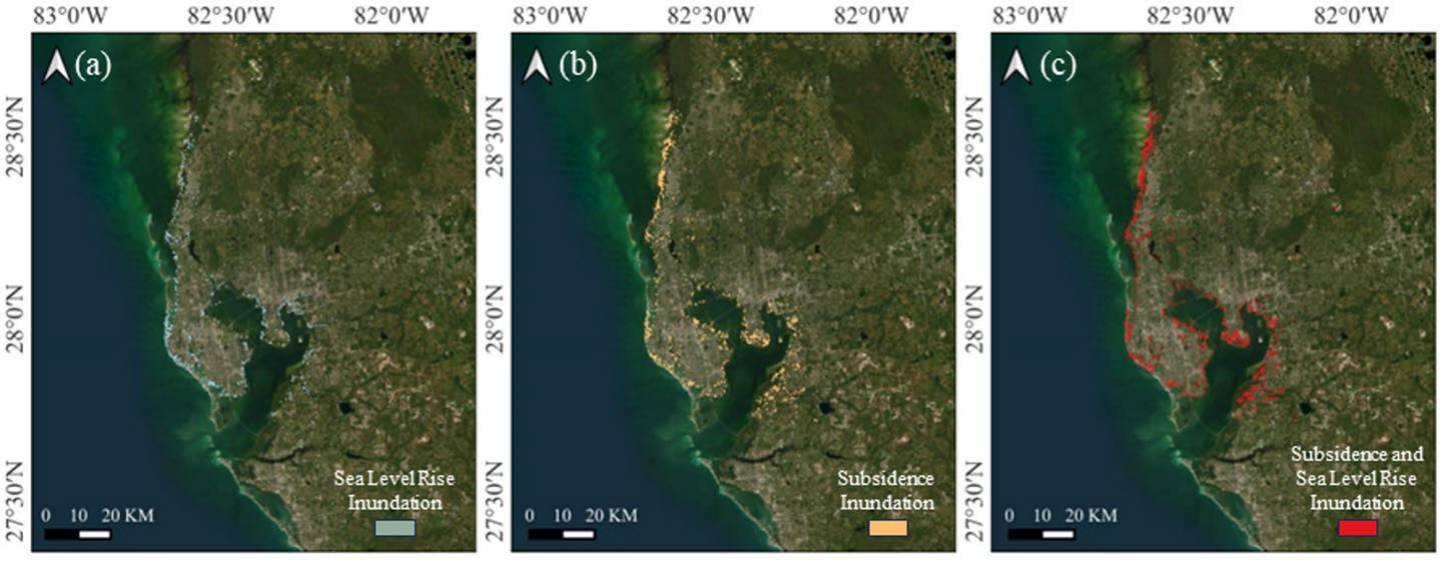
Inundation area in Tampa by 2100 due to **a** sea level rise, **b** subsidence, and **c** both sea level rise and subsidence

Tampa’s exposure to future flooding is relatively lower compared to other coastal cities (Fig. 20), largely due to its higher elevation and limited subsidence rates. However, sea level rise remains a critical concern. Under the SSP5-8.5 scenario, projections indicate a rise of 0.94 m by 2100, which would result in approximately 102.4 km^2^ of inundation. While much of Tampa sits at a higher elevation, its coastal regions, particularly those bordering Tampa Bay, remain vulnerable.

Although land subsidence in Tampa is generally lower than in other subsiding coastal regions, it contributes to the city’s overall flood exposure. In certain coastal zones, including areas near Tampa Bay and the stretch from St. Pete Beach to Hernando Beach, higher subsidence rates are experienced, ranging from 1 to 10 mm per year. Many of these locations are built on reclaimed land or soft, unconsolidated sediments, making them more prone to compaction. As a result, the land surface gradually lowers, effectively accelerating the relative rise of sea levels and exacerbating flood hazards in these areas.

When considering the effects of sea level rise and subsidence together, the total projected inundation area expands to 207.6 km^2^ by 2100. While this number is lower than that of other major coastal cities, it still indicates a substantial increase in flood-prone areas, particularly in low-lying coastal zones. These findings highlight the need for proactive flood mitigation measures, including improved coastal defenses and sustainable land-use planning, to address the growing risks in Tampa’s most vulnerable regions.

### Cross-city comparison of subsidence-related flood exposure

**Fig. 21:**
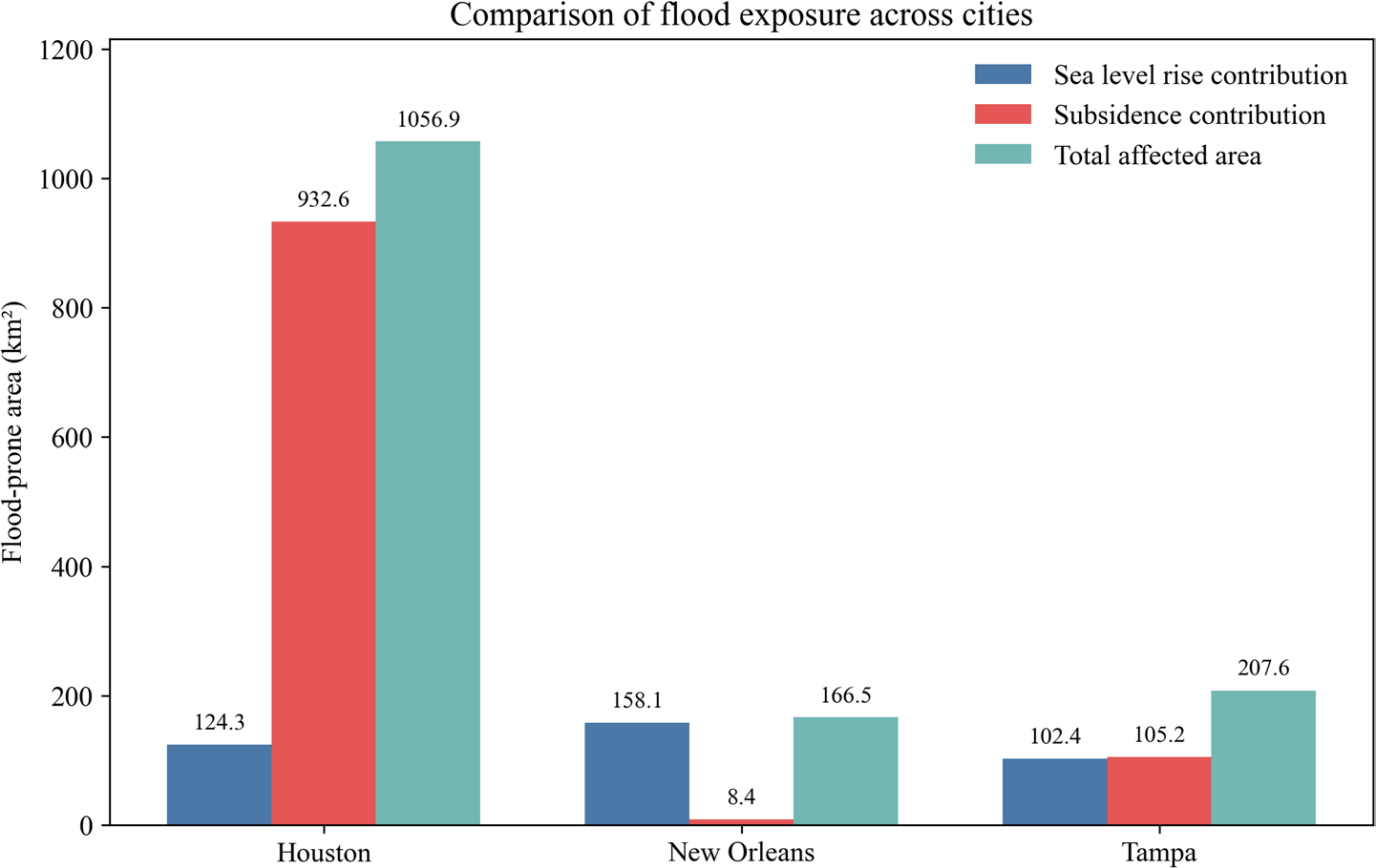
Comparison of flood exposure across cities

A comparison of the three coastal cities shows that the influence of subsidence varies substantially, even under the same sea-level-rise scenario (Fig. 21). In Houston, the additional area associated with subsidence is far larger than that exposed to sea-level rise alone. In practice, this means that flood-prone areas are not only intensified near the coastline but also extend inland across low-lying parts of the coastal plain. This has implications for planning, as areas that are not traditionally treated as coastal may still become increasingly exposed over time.

Conditions in New Orleans are different. The baseline exposure under sea-level rise is already extensive, and the additional area introduced by subsidence is comparatively small. Instead of expanding the footprint of risk, subsidence mainly deepens existing exposure. In this setting, the more immediate concern is not where flooding may newly occur, but how existing protection systems, particularly levees and pumping infrastructure, can continue to perform under progressively lower ground elevations.

Tampa does not follow either of these patterns exactly. The overall exposed area is smaller, but the additional impact of subsidence is still evident, especially along low-elevation coastal margins. The response here appears to be more sensitive to local topographic conditions, where relatively small elevation changes can shift areas across the flooding threshold. As a result, the effects are more spatially concentrated rather than widespread, suggesting that targeted interventions in sensitive areas may be more effective than broad regional measures.

These differences suggest that the role of subsidence in shaping flood risk depends strongly on local conditions. The same amount of ground lowering does not translate into the same type of exposure across different systems, and mitigation strategies should be adapted to local conditions. The results provide a basis for prioritizing planning and infrastructure investment by identifying where subsidence-driven changes in flood exposure are most significant.

## Conclusion

In this study, we utilize the SBAS-InSAR technique to develop a new land subsidence dataset for coastal cities in the Gulf of Mexico, focusing on Houston, New Orleans, and Tampa from 2019 to 2024. The results indicate significant subsidence, with velocities reaching − 42 mm/year in Houston, − 48 mm/year in New Orleans, and − 144 mm/year in Tampa. We identify several previously unrecognized deformation patterns, including subsidence in Liverpool (Houston), Jefferson Parish (New Orleans), and the mining region in Tampa. The quantitative analysis based on GWR shows that the influence of subsurface factors varies across regions. Groundwater-related processes show stronger and more consistent relationships with subsidence, particularly in Houston and Tampa, while hydrocarbon-related effects are generally more localized. In New Orleans, subsidence appears to be influenced by multiple factors, with more spatially variable relationships. By integrating InSAR-derived deformation data with projected sea level rise under the SSP5-8.5 scenario, we assess future flood risks in the coastal cities. The findings highlight that subsidence significantly exacerbates coastal flooding, particularly in Houston, where the total inundated area is projected to reach 1056.9 km^2^. New Orleans, due to its low elevation, is expected to experience 166.5 km^2^ of inundation, while Tampa is projected to have 207.6 km^2^ of inundation by 2100. More importantly, subsidence alters the spatial distribution of flood exposure, with distinct patterns observed across the three cities. These findings highlight the importance of incorporating land subsidence into flood risk assessment and provide a more realistic basis for regional-scale hazard evaluation and urban planning.
